# Differences in Contractile Function of Myofibrils within Human Embryonic Stem Cell-Derived Cardiomyocytes vs. Adult Ventricular Myofibrils Are Related to Distinct Sarcomeric Protein Isoforms

**DOI:** 10.3389/fphys.2017.01111

**Published:** 2018-01-19

**Authors:** Bogdan Iorga, Kristin Schwanke, Natalie Weber, Meike Wendland, Stephan Greten, Birgit Piep, Cristobal G. dos Remedios, Ulrich Martin, Robert Zweigerdt, Theresia Kraft, Bernhard Brenner

**Affiliations:** ^1^Department of Molecular and Cell Physiology, Hannover Medical School, Hannover, Germany; ^2^Department of Physical Chemistry, Faculty of Chemistry, University of Bucharest, Bucharest, Romania; ^3^Department of Cardiac, Thoracic, Transplantation and Vascular Surgery, Leibniz Research Laboratories for Biotechnology and Artificial Organs, REBIRTH-Center for Regenerative Medicine, Hannover Medical School, Hannover, Germany; ^4^Department of Anatomy, Bosch Institute, University of Sydney, Sydney, NSW, Australia

**Keywords:** human pluripotent stem cell-derived cardiomyocytes, human embryonic stem cell-derived cardiomyocytes, hiPSC-CMs maturation, human cardiomyocytes development, adult human ventricular myofibrils, cardiac contractile function, β-myosin isoform cross-bridge, cross-bridge kinetics

## Abstract

Characterizing the contractile function of human pluripotent stem cell-derived cardiomyocytes (hPSC-CMs) is key for advancing their utility for cellular disease models, promoting cell based heart repair, or developing novel pharmacological interventions targeting cardiac diseases. The aim of the present study was to understand whether steady-state and kinetic force parameters of β-myosin heavy chain (βMyHC) isoform-expressing myofibrils within human embryonic stem cell-derived cardiomyocytes (hESC-CMs) differentiated *in vitro* resemble those of human ventricular myofibrils (hvMFs) isolated from adult donor hearts. Contractile parameters were determined using the same micromechanical method and experimental conditions for both types of myofibrils. We identified isoforms and phosphorylation of main sarcomeric proteins involved in the modulation of force generation of both, chemically demembranated hESC-CMs (d-hESC-CMs) and hvMFs. Our results indicate that at saturating Ca^2+^ concentration, both human-derived contractile systems developed forces with similar rate constants (0.66 and 0.68 s^−1^), reaching maximum isometric force that was significantly smaller for d-hESC-CMs (42 kPa) than for hvMFs (94 kPa). At submaximal Ca^2+^-activation, where intact cardiomyocytes normally operate, contractile parameters of d-hESC-CMs and hvMFs exhibited differences. Ca^2+^ sensitivity of force was higher for d-hESC-CMs (pCa_50_ = 6.04) than for hvMFs (pCa_50_ = 5.80). At half-maximum activation, the rate constant for force redevelopment was significantly faster for d-hESC-CMs (0.51 s^−1^) than for hvMFs (0.28 s^−1^). During myofibril relaxation, kinetics of the slow force decay phase were significantly faster for d-hESC-CMs (0.26 s^−1^) than for hvMFs (0.21 s^−1^), while kinetics of the fast force decay were similar and ~20x faster. Protein analysis revealed that hESC-CMs had essentially no cardiac troponin-I, and partially non-ventricular isoforms of some other sarcomeric proteins, explaining the functional discrepancies. The sarcomeric protein isoform pattern of hESC-CMs had features of human cardiomyocytes at an early developmental stage. The study indicates that morphological and ultrastructural maturation of βMyHC isoform-expressing hESC-CMs is not necessarily accompanied by ventricular-like expression of all sarcomeric proteins. Our data suggest that hPSC-CMs could provide useful tools for investigating inherited cardiac diseases affecting contractile function during early developmental stages.

## Introduction

*In vitro* differentiation of human pluripotent stem cells (hPSCs) toward cardiomyocytes provides a model system of human cardiac myogenesis (Murry and Keller, 2008; Birket and Mummery, 2015; Kempf et al., 2016b). Given the in principal unlimited availability of human embryonic (hESC) and induced pluripotent stem cells (hiPSC) (Kempf et al., 2016a), hPSC-derived cardiomyocytes also hold great promise for the treatment of cardiovascular diseases by cell transplantation or engineered cardiac tissue (Kensah et al., 2013; Zimmermann, 2017), for assessing efficiency and toxicity of pharmacological compounds (Burridge et al., 2016), or to be used as cellular disease models *in vitro* (Moretti et al., 2013). Particularly, cardiomyocytes (CMs) derived from patient-specific hPSCs have facilitated studies of the consequences of hereditary diseases *in vitro* (Jung and Bernstein, 2014; Kamdar et al., 2015; Pioner et al., 2016). For instance, in hypertrophic cardiomyopathy (HCM), mutations of specific sarcomeric proteins can impair the contractile function of CMs. The related abnormal changes in force generation of CMs may subsequently result in disorder of other cellular functions and further pathological alterations (Kraft et al., 2013; Brenner et al., 2014; Fatkin et al., 2014). Detailed analysis of the contractile function of hPSC-CMs is therefore crucial to further characterize hPSC-CMs as model systems for investigation of disease mechanisms and future therapies.

Whether the contractile function of myofibrils within hPSC-CMs matches that of ventricular myofibrils determining the pump function in the adult human heart is an important question. Previous studies have shown that hPSC-CMs contract periodically, thus revealing the presence of functional myofibrils, which are typically less well-aligned compared to adult human CMs (Bedada et al., 2014; Jung and Bernstein, 2014; Yang et al., 2014a; Pioner et al., 2016; Weber et al., 2016). Achieving more matured hPSC-CMs seems favorable for the *in vitro* modeling of pathological conditions of the adult heart. On the other hand, an ongoing maturation of early hPSC-CMs *in vitro* may support molecular and cellular investigations on the disease onset at developmental stages. In this view, very little is known about the *in utero* onset of cardiomyopathies (Fatkin et al., 2014) and congenital heart diseases (Elhamine et al., 2016), as well as the demise of fetuses and neonates survival in such conditions (Mongiovì et al., 2010; MacColl et al., 2014).

The contractile function in muscle cells is driven by the acto-myosin interaction, where cross-bridges cycle between strongly bound, force-generating and weakly bound, non-force generating conformations (Huxley, 1957; Brenner, 1991a). Kinetic parameters of the force generated by sarcomeres depend mainly on the myosin isoform. Usually, after typical differentiation protocols, sarcomeres of hPSC-CMs are composed of a mixture of α and β isoforms of the myosin heavy chain (MyHC) (Weber et al., 2016), while in the adult, neonate and developing human ventricle the βMyHC isoform predominates (Bouvagnet et al., 1987; Miyata et al., 2000; Reiser et al., 2001; Elhamine et al., 2016; Racca et al., 2016). In addition, most known HCM-related myosin-mutations are expressed in the βMyHC rather than in the αMyHC isoform (Fatkin et al., 2014; Burke et al., 2016). We recently showed that long-term cultivation of human embryonic stem cell-derived cardiomyocytes (hESC-CMs) on stiff isotropic substrates (e.g., laminin-coated glass cover-slips) shifts myosin expression exclusively toward the βMyHC isoform (Weber et al., 2016).

In the present study, we addressed the question whether βMyHC-expressing myofibrils within hESC-derived CMs have the same contractile properties as adult human ventricular myofibrils (hvMFs). Therefore, we aimed to identify which steady-state and kinetic force parameters of myofibrils within hESC-CMs differentiated *in vitro* resemble the corresponding force parameters generated by adult hvMFs. To directly assess the contractile function of myofibrillar bundles of single cells, chemically demembranated hESC-CMs (d-hESC-CMs) were used. For comparison with healthy human heart, small hvMFs-bundles were isolated from chemically demembranated tissues of adult donor hearts. Biomechanical assessment of such myofibrils at defined Ca^2+^ concentrations ([Ca^2+^]) allows direct examination of the cycling cross-bridge-driven contractile performance of myofibrils, including relaxation kinetics, in the absence of Ca^2+^ handling systems and of upstream signaling modulated by hormonal activities (Poggesi et al., 2005; Stehle et al., 2009; Walker et al., 2011). We measured steady-state and kinetic parameters of isometric forces generated by myofibrils of d-hESC-CMs and by hvMFs using the same micromechanical method and experimental conditions (Stehle et al., 2002b; Weber et al., 2016), and characterized the isoforms and phosphorylation status of main sarcomeric proteins involved in modulation of force generation. Observed similarities and subtle differences in force kinetics are discussed using modeling based on the cross-bridge theory (Huxley, 1957; Brenner, 1988, 1990, 1991a; Brenner and Chalovich, 1999).

## Materials and methods

### Solutions composition

Composition of solutions used to prepare d-hESC-CMs and hvMFs (*Na-solution, K-solution, pCa* > *9, pH 7.0*), protease inhibitor cocktail (PIC) and solutions used to activate (*Ca*^*2+*^*-activating-solution, pCa 4.18, pH 7.1*) and relax (*Relaxing-solution, pCa* > *8, pH 7.1*) myofibrils for the assessment of their contractile function was described in Weber et al. (2016). Ca^2+^-activating solutions at intermediate [Ca^2+^] were obtained by mixing the relaxing and activating solutions in the appropriate ratio as in Kraft et al. (2013).

### Preparation of d-hESC-CMs and hvMFs

#### Demembranated human embryonic stem cell-derived cardiomyocytes (d-hESC-CMs)

Experimental details regarding differentiation and enrichment of hESC-CMs in 12–20 days of suspension culture using defined differentiation media supplemented with Wnt-pathway modulators were previously published (Kempf et al., 2014) (see Supplementary Material). Such suspension culture-derived “cardiac bodies” consisting of essentially pure hESC-CMs were enzymatically dissociated and ~118 cells/mm^2^ were plated for long-term cultivation on laminin-coated glass cover-slips. This resulted in a majority of hESC-CMs expressing essentially only the βMyHC isoform at the protein level (Weber et al., 2016). We used hESC-CMs from three different cell batches. In this study, hESC-CM (cf. online Supplementary Material, Video 1) were used after 35–56 days of cultivation on glass cover-slips. hESC-CMs were chemically demembranated using 0.5% Triton-X-100 in the presence of 20 mM BDM as previously described (Weber et al., 2016), and then equilibrated with *relaxing-solution* containing PIC and 5 mM DTT (without BDM) before the experiment.

#### Human ventricular myofibrils (hvMFs)

hvMFs were isolated from two different non-transplanted donor hearts for which no suitable recipient was found. Long-term cryopreservation of muscular tissues was previously described (Kraft et al., 2013). After quick thawing in *Na-solution* including 20 mM BDM (~20°C), ventricular fragments were cut in small pieces removing connective tissues and fat traces in cold *Na-solution* (+BDM), and then demembranated in cold *K-solution* containing 0.5% Triton-X-100 and 20 mM BDM (~50 min, 5°C). Detergent was rinsed twice using cold *K-solution* without Triton (~50 min). hvMFs-bundles were prepared freshly before the micromechanical experiments by homogenizing the demembranated ventricular pieces equilibrated with *relaxing-solution* (+PIC, +DTT 5 mM, on ice) for 5–10 s with a blender (Ultra-Turrax T8, IKA Labortechnik, Germany) at 25,000 rpm. The resulting myofibrillar suspension was filtered through a mesh (pore size 21 μm) to remove the thick myofibrillar aggregates. The homogenate was further centrifuged (400 × g, 10 min, 5°C; Biofuge Primo-R, Thermo-Fisher, Massachusetts, USA) and the pellet re-suspended in fresh *relaxing-solution* (+PIC, +DTT) or subjected to further treatments.

Approval of Hannover Medical School Ethics Committee was obtained for anonymized use of the different muscle biopsies and the hESC-CMs in our study (approval numbers 2729–2001, 507–2009, 1751–2013). All subjects gave written informed consent at the different institutions in accordance with the Declaration of Helsinki.

### PKA treatment, immunostaining, and analysis of sarcomeric proteins

Details regarding PKA-treatment, double and single immunostaining, and analysis of sarcomeric proteins of d-hESC-CMs and hvMFs are described in Supplementary Material.

### Micromechanical investigations

Basic features and technical details of the custom-built micromechanical setup used to assess the contractile function of either single d-hESC-CMs or small hvMFs-bundles were previously described (Colomo et al., 1998; Stehle et al., 2002a,b; Weber et al., 2016).

#### hvMFs

Three to five hundred microliters of myofibrillar homogenate in *relaxing-solution* was pipetted in the pre-cooled chamber of the micromechanical setup and myofibrils allowed to sediment for ~1 h. Then the chamber was filled with *relaxing-solution* (15°C).

#### d-hESC-CMs

A laminin-coated glass cover-slip containing plated, chemically demembranated d-hESC-CMs was transferred into the chamber of the micromechanical setup, previously filled with *relaxing-solution* (15°C).

Contractile function of a single d-hESC-CM containing mainly few bundled myofibrils or a small hvMFs-bundle was investigated (Videos 2–4) with a custom-built micromechanical setup mounted on an inverted microscope (Olympus IX-71). Experiments with d-hESC-CMs and hvMFs were performed with solutions from the same batch at 15°C. Working at this temperature allows comparison of the functional results to several biomechanical studies with hvMFs or skinned cardiomyocytes which were previously performed at 15°C (Stehle et al., 2002b; van der Velden et al., 2003b; Piroddi et al., 2007; Walker et al., 2011; Kraft et al., 2013; van Dijk et al., 2014; Pioner et al., 2016; Racca et al., 2016). Some details about the micromechanical setup are described in Supplementary Material.

**Figure 1:**
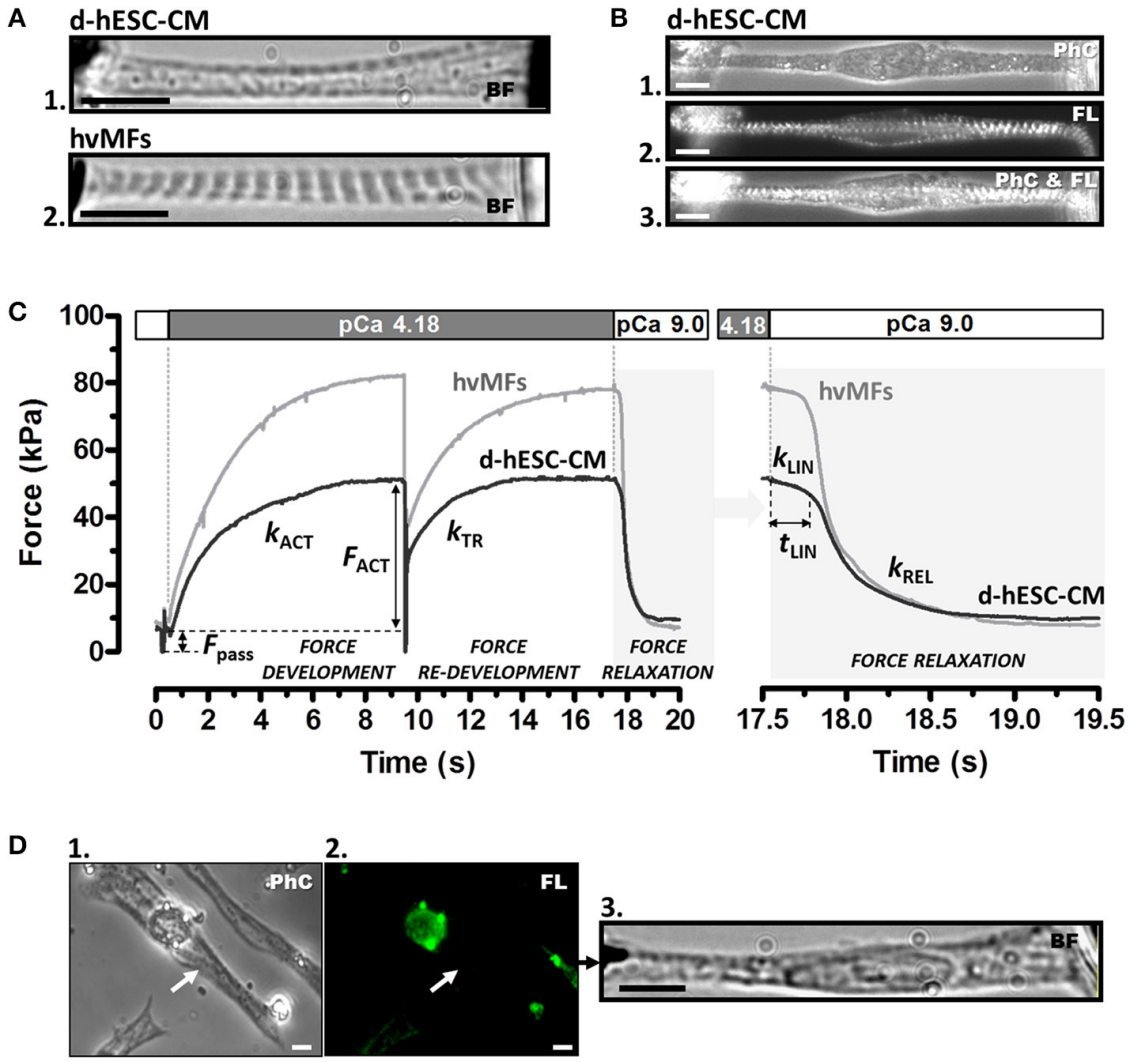
**(A)** Contractile function of myofibrils of a single demembranated human stem cell-derived cardiomyocyte (d-hESC-CM) **(A**_1_**)** and contractile function of a single bundle of adult human ventricular myofibrils (hvMFs) **(A**_2_**)** were investigated using the same micromechanical setup and experimental conditions. Microphotographs **(A**_1_**,A**_2_**)** were taken in bright field (BF). **(B)** A single elongated d-hESC-CM immunostained against α-actinin was observed in phase contrast (PhC; **B**_1_), fluorescence (FL; **B**_2_), or simultaneously in PhC and FL **(B**_3_**)**. Note the predominant axial alignment of myofibrils within d-hESC-CMs **(A**_1_**,B**_1−3_**)**. **(C)** Representative force transients of a single d-hESC-CM (black trace; d-hESC-CMs shown in A_1_ and Video 3) and of a hvMFs-bundle (gray trace; hvMFs-bundle shown in **A**_2_ and Video 4) mounted and held isometrically between the tips of a stiff needle (left) and a nN-sensitive force probe (right). Isometric active (*F*_ACT_) at saturating [Ca^2+^] (pCa 4.18) and passive (*F*_pass_) forces, rate constant *k*_ACT_ of Ca^2+^-induced force development, rate constant *k*_TR_ of force re-development and the three kinetic parameters (*k*_LIN_, *t*_LIN_, *k*_REL_) characterizing force relaxation upon rapid Ca^2+^-removal (detailed in the right panel) are indicated for this d-hESC-CM (T = 15°C, pCa 4.18): *F*_ACT_ = 43 kPa, *F*_pass_ = 7 kPa, *k*_ACT_ = 0.60 s^−1^, *k*_TR_ = 0.65 s^−1^, *k*_LIN_ = 0.30 s^−1^, *t*_LIN_ = 243 ms, *k*_REL_ = 4.2 s^−1^; and for the single hvMF-bundle: *F*_ACT_ = 72 kPa, *F*_pass_ = 9 kPa, *k*_ACT_ = 0.50 s^−1^, *k*_TR_ = 0.56 s^−1^, *k*_LIN_ = 0.23 s^−1^, *t*_LIN_ = 216 ms, *k*_REL_ = 5.3 s^−1^. **(D)** d-hESC-CM (indicated by arrow in **D**_1_) immunostained solely against αMyHC isoform and exhibiting no sarcomeric fluorescence (i.e., βMyHC-positive d-hESC-CM; **D**_2_) was micromechanically investigated **(D**_3_**)**. Force parameters from several immunostained d-hESC-CMs are given in Table 1. Images were taken in PhC **(D**_1_**)**, FL **(D**_2_**)**, or BF **(D**_3_**)**. All scale bars = 10 μm.

Sarcomere length (SL) and cross-sectional area (CSA) of myofibrils within d-hESC-CMs and of hvMF-bundles were determined in bright field (BF) or phase contrast (PhC) at 96-fold magnification (Figures 1A,B), using a CCD camera (Hamamatsu Photonics, Herrsching am Ammersee, Germany) attached to microscope. To calculate specific forces (force/CSA, kPa = nN/μm^2^) the mean diameter of the myofibrils or myofibrillar bundles within d-hESC-CMs and of entire hvMF-bundles was assessed prior to force measurements as previously described (Stehle et al., 2002b; Weber et al., 2016). In particular, for d-hESC-CMs total CSA of all myofibrils observed within the demembranated cells as darker traces on brighter background (Figure 1A_1_) was calculated assuming a cylindrical shape of the myofibrils. As a control for myofibrillar content and diameter in d-hESC-CMs, some cells were immunostained against α-actinin prior to Ca^2+^-activation (Figures 1B_1−3_), as previously described for isolated myofibrils (Telley et al., 2006). This allowed visualizing myofibrillar thickness and distribution within the cardiomyocytes and to further compare these features with the corresponding ones assigned to the darker traces observed either in PhC (e.g., Figures 1B_1−3_) within the same cardiomyocyte or in BF (e.g., Figure 1A_1_). Such analysis suggested essentially the same thickness for the fluorescently labeled myofibrils at their Z-disks and for the dark fascicles within d-hESC-CMs (in BF or PhC). This is also indicated by Video 5 where visualization of sarcomeres by PhC, fluorescence (FL), or overlapped (PhC and FL) is shown. Based on this we measured myofibrillar CSA directly from the dark fascicles in BF or PhC, and thus, reduced the overestimation of CSA which occurs if we would take the total width of the mounted d-hESC-CMs. An overestimated myofibrillar CSA would yield smaller specific force values.

### Considerations based on the two-state cross-bridge model

For the interpretation of steady-state and kinetic force parameters emerging from micromechanical measurements with d-hESC-CMs and hvMFs (see “Discussion”), we used a Huxley-Brenner two-state model (Huxley, 1957; Brenner, 1988, 1990, 1991a,b; Brenner and Chalovich, 1999). During muscle activation, one state represents cross-bridges in strongly-binding configurations, while the other state corresponds to cross-bridges in weakly-binding configurations. Each of the two states accommodates many intermediate cross-bridge sub-states of the myosin ATPase reaction pathway. The apparent rate constant *f*_app_ describes the probability of cross-bridge transition from the non-force generating (weakly-binding) states to the force-generating (strongly-binding) states upon phosphate release. The apparent rate constant *g*_app_ describes the probability of the opposite transition of cross-bridges from the force-generating to the non-force generating states via ADP-release and ATP-binding steps. Both transitions occur through dynamic equilibria of cross-bridges attachment/detachment (Brenner, 1991b) (e.g., cross-bridge detachment does not rate-limit *g*_app_). Probability *f*_app_ is dependent on the Ca^2+^-regulated thin filament status (i.e., modulated by the variation of [Ca^2+^]), while the probability *g*_app_ is independent of [Ca^2+^] (a first-order rate constant). Both parameters may influence isometric force response, i.e., regulation of the steady-state force response through turnover kinetics (Brenner, 1988). The measured rate constant of force re-development *k*_TR_ (see below) is given by *f*_app_+*g*_app_ (Brenner, 1988, 1990, 1991a). The measured rate constant *k*_LIN_ of the slow force decay during the first phase of the myofibrillar relaxation (see below) allows estimation of *g*_app_ (Stehle et al., 2002a,b; Poggesi et al., 2005).

### Force measurements and cross-bridge kinetics

d-hESC-CMs and hvMFs in relaxing solution were pre-stretched to *SL* = 2.3 μm from their different individual slack *SL* (*SL*_0_). Then, a slack-restretch maneuver (amplitude 30% of the length of pre-stretched myofibrils) was applied to cancel transiently the passive force (*F*_pass_) stored in the elastic elements of myofibrils. Therefore, this maneuver allows determination of *F*_pass_. The passive stretch further increased the parallel arrangement of myofibrils within the elongated d-hESC-CMs.

A sudden change to *Ca*^*2+*^*-activating-solution* with defined [Ca^2+^] determined the force to rise mono-exponentially in both d-hESC-CMs and hvMFs (Figure 1C) with the rate constant *k*_ACT_ (rate constant of Ca^2+^-induced force development), which depends on Ca^2+^-induced thin filament activation, cross-bridge formation and force generating conformational change, and cross-bridge turnover. When the developing force reached the maximum steady-state level, a quick (50 ms) release-restretch maneuver (amplitude 30% of the length of pre-stretched myofibrils) was applied which mechanically unloads the contracted myofibrils. The quick release allows a redistribution of the cross-bridges in the non-force-generating states before restretch and force re-development. This maneuver enables, at a given [Ca^2+^], the measurement of the total generated isometric force and of the rate constant of force re-development *k*_TR_ (rate constant of mechanically-induced force re-development) re-approaching steady-state force level, while the thin filament is already Ca^2+^-activated (Figure 1C) (Brenner, 1988). *k*_TR_ reflects the kinetics of the cross-bridges re-distribution from the non-force- to the force-generating states. Passive force (*F*_pass_) was subtracted from the recorded total force values (Figure 1C), thus yielding the isometric force (*F*_ACT_) generated by cycling cross-bridges.

Upon rapidly switching back to *relaxing-solution*, d-hESC-CMs and hvMFs undergo a biphasic force relaxation behavior (Figure 1C-right panel): first, force decays slowly and almost linearly during the period *t*_LIN_ with the rate constant *k*_LIN_ (early relaxation phase) (Stehle et al., 2002b; Stehle and Iorga, 2010). In the second, mono-exponential relaxation phase, force drops faster with the rate constant *k*_REL_ (late relaxation phase; Videos 2–4). Observations of the video streams immediately after rapid Ca^2+^ removal revealed that *SL* of d-hESC-CMs and hvMFs remained quasi-constant during the first (slow) relaxation phase, whereas the second (fast) relaxation phase was dominated by the dynamics of the sarcomeres, as previously described (Stehle et al., 2002b; Stehle and Iorga, 2010). Therefore, *k*_LIN_ is the rate constant determined by strained cross-bridges leaving the force-generating states during the isometric condition at sarcomeric level, while *k*_REL_ is the rate constant of less strained remaining cross-bridges leaving the force-generating states during lengthening of the sarcomeres along the myofibrils (Stehle et al., 2002a,b; Poggesi et al., 2005).

### Data analysis and statistics

The trace of force rise after activation and after quick release-restretch of the myofibrils was fitted by a mono-exponential function yielding *k*_ACT_ and *k*_TR_, respectively. For the relaxation, force decay was fitted by a function consisting of a linear and mono-exponential term yielding the parameters *k*_LIN_, *t*_LIN_, and *k*_REL_ of the force relaxation kinetics (Stehle et al., 2002b). Ca^2+^ concentration was given as pCa = −log_10_[Ca^2+^]. Data of normalized force-pCa relation, resulting from each measured sample, was fitted by the Hill-type equation, where *F*_n_ is the fractional force at a given pCa. *F*_n_ = *F*_ACT_/*F*_ACT,max_ where *F*_ACT,max_ is the maximum force recorded at pCa 4.18 (*F*_n_ = 1), pCa_50_ (indicating Ca^2+^-sensitivity of force) is the pCa at half-maximum force (*F*_n_ = 0.5), and *n*_H_ is the steepness of the force-pCa relation (Hill-coefficient):

$$\begin{array}{l} F_{n}\left(pCa\right)=\frac{1}{1+10^{n_{H}\left(pCa_{50}-pCa\right)}}. \end{array}$$

If not stated otherwise, steady-state and kinetic parameters were indicated as *mean* ± *SD* (Standard Deviation). Mean values were compared using unpaired Student *t*-test with significant differences when *p* < 0.05 (^*^), *p* < 0.01 (^**^), or *p* < 0.001 (^***^).

From each donor ventricle 29 hvMFs-bundles were investigated for their contractile function (–PKA: 14 and 11 bundles, +PKA: 15 and 18 bundles, respectively), and data of hvMFs were pooled together.

## Results

### Micromechanical investigations of d-hESC-CMs vs. hvMFs reveal differences of their sarcomeric contractile function at submaximal Ca^2+^-activation levels

The main function of cardiomyocytes is to generate force and shorten their length, thus determining the pump function of the heart. Therefore, we focused on the function of the contractile elements of the cardiomyocytes, the myofibrils. Our main aim was to identify whether steady-state and kinetic force parameters of myofibrils within d-hESC-CMs resemble the corresponding force parameters of hvMFs.

We used elongated demembranated hESC-CMs (e.g., Figure 1A_1_) enabling measurements of the force generated predominantly axially. Single d-hESC-CMs had as mean diameter of their myofibrillar-bundles *d* = 2.0-5.0 μm, length was *L* = 30-100 μm, slack sarcomere length was *SL*_0_ = 1.75–2.10 μm, and hvMFs-bundles (e.g., Figure 1A_2_) had *d* = 1.9–5.8 μm, *L* = 20–50 μm, and *SL*_0_ = 1.8–2.0 μm. Both d-hESC-CMs and hvMFs were initially exposed to *relaxing-solution* at 15°C and pre-stretched to *SL* = 2.3 μm. The comparison of the resulting passive forces (*F*_pass_) stored in the elastic elements of myofibrils in relaxing solution suggested that d-hESC-CMs (*F*_pass_ = 9 ± 3 kPa, *n* = 12) were, in average, more compliant (*p* < 0.0001) than hvMFs (*F*_pass_ = 18 ± 7 kPa, *n* = 25) (Figure S1). *F*_pass_ was not responsive (*p* = 0.712) to PKA-treatment for d-hESC-CMs (*F*_pass_ = 10 ± 4 kPa, *n* = 14; + PKA), but it was slightly reduced (*p* = 0.029) for hvMFs from 18 ± 7 kPa (*n* = 25; −PKA) to 15 ± 4 kPa (*n* = 33; + PKA) (Figure S1).

#### Steady-state and kinetic force parameters at saturating [Ca^2+^]

When activated at saturating [Ca^2+^], kinetics of force development (*k*_ACT_) and re-development (*k*_TR_) of d-hESC-CMs (*k*_ACT_ = 0.66 ± 0.14 s^−1^, *n* = 11; *k*_TR_ = 0.74 ± 0.10 s^−1^, *n* = 11) and hvMFs (*k*_ACT_ = 0.68 ± 0.14 s^−1^, *n* = 37; *k*_TR_ = 0.68 ± 0.10 s^−1^, *n* = 37) were similar, respectively, with values as previously reported for hvMFs (Stehle et al., 2002b; Piroddi et al., 2007; Walker et al., 2011; Racca et al., 2016). Maximum isometric force (*F*_ACT,max_) for d-hESC-CMs (*F*_ACT,max_ = 42 ± 10 kPa, *n* = 12) was significantly (*p* < 0.001) smaller than the *F*_ACT,max_ generated by hvMFs (*F*_ACT,max_ = 94 ± 25 kPa, *n* = 39) (Figure 1C).

However, we cannot exclude the presence of a very low amount of residual αMyHC in d-hESC-CMs [however, under detection limit in gel analysis; see section Overview of Sarcomeric Proteins by Gel Electrophoresis]. To exclude such CMs from the functional analysis, some d-hESC-CMs were immunostained against αMyHC prior micromechanical measurements. Then, *F*_ACT,max_, *k*_TR_, and *k*_LIN_ were determined at pCa 4.18 for d-hESC-CMs exhibiting null fluorescence, i.e., βMyHC positive cells which had no αMyHC-fluorescence (Figure 1D). These values were taken as reference values for all d-hESC-CMs that were characterized micromechanically without preceding immunostaining (Table 1). It has been shown previously that *k*_TR_ of d-hESC-CMs which also have αMyHC-positive sarcomeres is significantly faster than for purely βMyHC-positive CMs (Weber et al., 2016). Here we included all d-hESC-CMs measured without preceding immunostaining which had individual *k*_TR_ and *k*_LIN_ values that were smaller than the upper limit set for *k*_TR_ to 0.87 s^−1^ (= *mean* + 2 × *SD* = 0.70 + 0.17) and for *k*_LIN_ to 0.47 s^−1^ (= *mean* + 2 × *SD* = 0.29 + 0.18), respectively (Table 1). Therefore, there is a 95% probability that d-hESC-CMs included in the analysis without preceding immunostaining are also negative for αMyHC.

**Table 1:** Force kinetic parameters (*k*_TR_, *k*_LIN_) and maximum isometric force (*F*_ACT,max_) generated at saturating Ca^2+^ concentration (pCa 4.18, 15°C) by d-hESC-CMs which were identified as purely βMyHC positive CMs by immunostaining against αMyHC.

**Force parameters**	**Immunostained d-hESC-CMs**	**Not immunostained d-hESC-CMs**
*F*_ACT,max_ (kPa)	39 ± 13	42 ± 10
*k*_TR_ (s^−1^)	0.70 ± 0.09	0.74 ± 0.10
*k*_LIN_ (s^−1^)	0.29 ± 0.09	0.26 ± 0.06
Number of cells	13	15
Plated (days)	37–56	36–50

*Data are compared to kinetic parameters of d-hESC-CMs which were measured without preceding immunostaining. Data shown as mean ± SD. There were no significant differences for the mean of each parameter determined between “immunostained” and “not immunostained” d-hESC-CMs; (p > 0.05, Student's unpaired t-test)*.

PKA-treatment did not significantly affect *F*_ACT,max_, *k*_ACT_, and *k*_TR_ force parameters at pCa 4.18 of either myofibrils within d-hESC-CMs (*F*_ACT,max_ = 37 ± 11 kPa, *n* = 15; *k*_ACT_ = 0.62 ± 0.13 s^−1^, *n* = 15; *k*_TR_ = 0.69 ± 0.11 s^−1^, *n* = 16) or hvMFs (*F*_ACT,max_ = 95 ± 27 kPa, *n* = 33; *k*_ACT_ = 0.64 ± 0.11 s^−1^, *n* = 33; *k*_TR_ = 0.67 ± 0.09 s^−1^, *n* = 33). This is consistent with previous observations using human ventricular myofibrils and skinned cardiomyocytes (Walker et al., 2011; Kraft et al., 2013).

The results show that at saturating [Ca^2+^], cycling βMyHC cross-bridges are able to develop force with similar rate constants, independent of PKA-treatment, for both contractile systems (d-hESC-CMs and hvMFs). Yet, considering the lower maximum force level reached by d-hESC-CMs, the rates by which both contractile systems reach maximum force were significantly different (*p* < 0.001), being slower for d-hESC-CMs (28.2 ± 8.8 kPa/s, *n* = 11) than for hvMFs (64.8 ± 26.9 kPa/s, *n* = 37).

#### Steady-state force response at intermediate [Ca^2+^]

**Figure 2:**
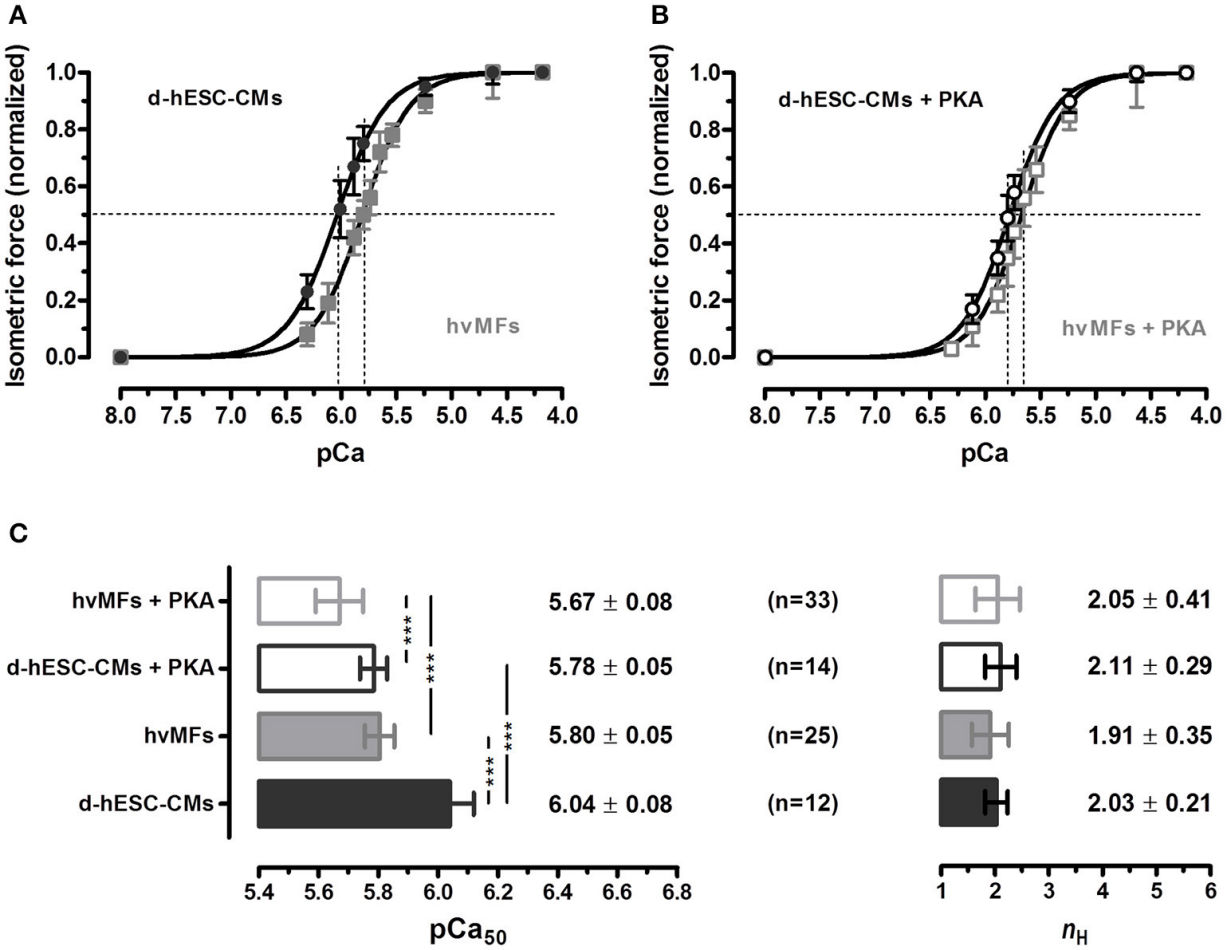
**(A,B)** Normalized isometric force response at different Ca^2+^ concentrations (given as pCa) of d-hESC-CMs (black circles and bars) and hvMFs (gray squares and bars), with (open symbols and bars) and without (filled symbols and bars) PKA-treatment prior to force measurements. Measured force at different pCa were normalized to the maximum force generated at saturating [Ca^2+^] (pCa 4.18; 15°C) and fitted by a dose-response Hill-type equation given in “Material and Methods.” **(C)** pCa at half maximum force generation (pCa_50_; indicating Ca^2+^-sensitivity of force), and the steepness of the force-pCa relationship *(n*_H_ or Hill coefficient). Data are given as mean ± *SD*; ^***^p < 0.001. *n*, number of d-hESC-CMs and hvMFs, respectively. Force parameters of hvMFs isolated from two different adult donor hearts were pooled together.

Cardiomyocytes *in vivo* operate at intermediate [Ca^2+^]. Therefore, it is important also to evaluate and compare myofibrillar contractile function of d-hESC-CMs and hvMFs at submaximal [Ca^2+^]. d-hESC-CMs responded to intermediate [Ca^2+^] with significantly (*p* < 0.001) higher isometric force when compared to hvMFs (ΔpCa_50_ = +0.24; Figure 2A). pCa determined at half of the maximum generated force (pCa_50_) was 6.04 ± 0.08 for d-hESC-CMs and 5.80 ± 0.05 for hvMFs (Figure 2C). The force-pCa curve determined for d-hESC-CMs had similar steepness (*n*_H_) as the curve determined for hvMFs (Figures 2A–C).

Phosphorylation levels of some sarcomeric proteins can persist even after chemical skinning of myocardial samples. In studies with human cardiomyocytes isolated from failing and non-failing hearts (van der Velden et al., 2003b), PKA-treatment shifted the force-pCa curves to the right-side. Here, we found that PKA-treatment also shifted the isometric force-pCa relationships of both d-hESC-CMs (ΔpCa_50_ = −0.26) and hvMFs (ΔpCa_50_ = −0.13) distinctly to higher [Ca^2+^], while the steepness (*n*_H_) of the curves was not significantly affected (Figure 2C).

#### Kinetics of the force rise to different Ca^2+^-dependent force levels

**Figure 3:**
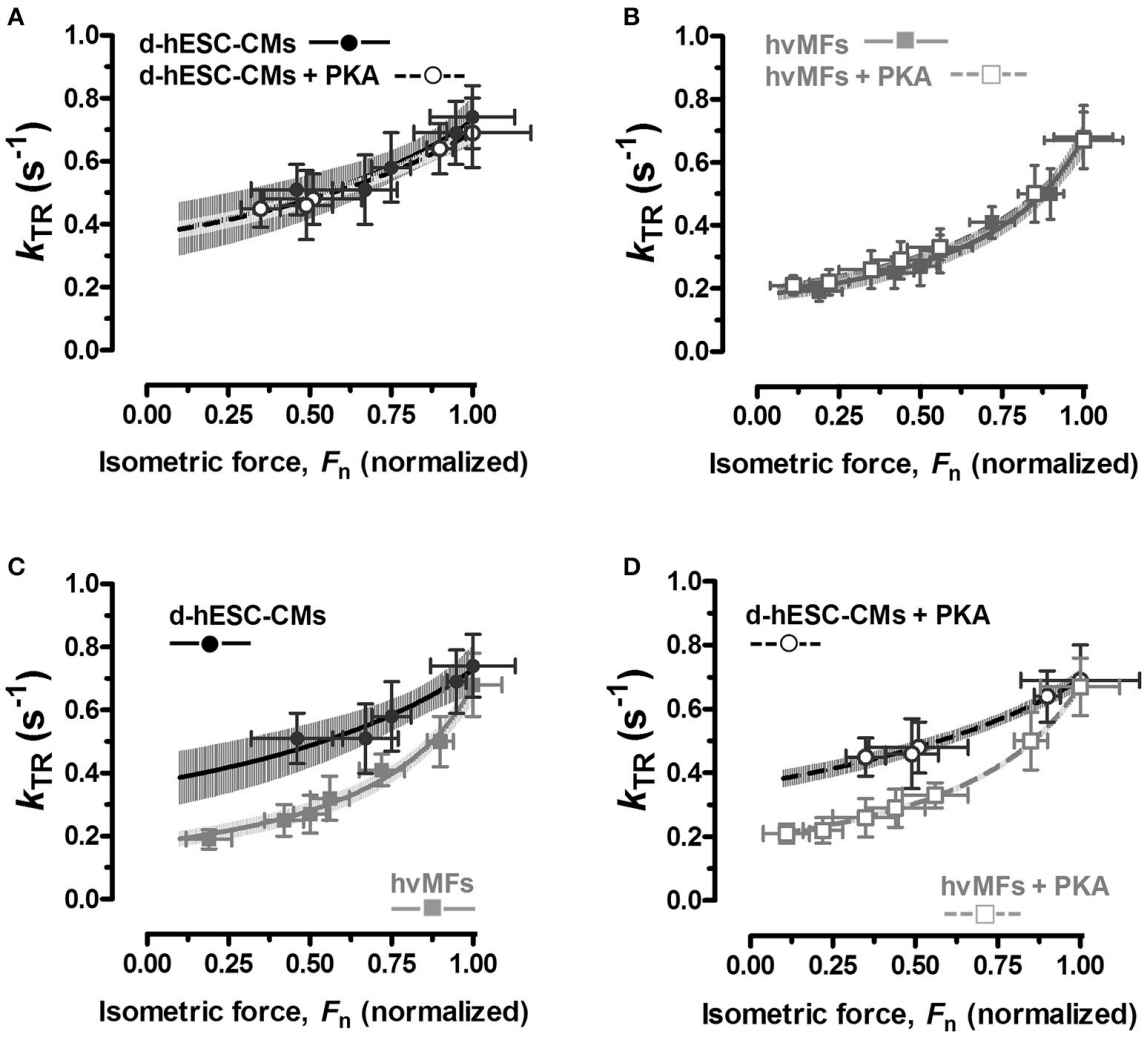
**(A–D)** Relationships between measured rate constants *k*_TR_ of force re-development at different [Ca^2+^] and the fractional isometric force (*F*_n_ = *F*_ACT_/*F*_ACT,max_) for d-hESC-CMs (black symbols) and hvMFs (gray symbols), with (open symbols), and without (closed symbols) PKA-treatment prior to measurements. The equation *k*_TR_ = *g*_app_/(1−*F*_n_·*f*_app,max_/(*f*_app,max_+*g*_app_)), which was used to fit relations of *k*_TR_ vs. *F*_n_ (Poggesi et al., 2005), is based on the two-state cross-bridge model (Huxley, 1957; Brenner, 1990). 95%-confidence intervals of the relations are shown, in distinct gray levels, for d-hESC-CMs ± PKA **(A)**, hvMFs ± PKA **(B)**, and for both d-hESC-CMs and hvMFs either not treated **(C)**, or treated **(D)** with PKA, respectively. *n*, number of d-hESC-CMs and hvMFs as in Figure 2C.

The force response at a given [Ca^2+^] (considering both absolute and relative values) was different in d-hESC-CMs than in hvMFs. We compared at different intermediate [Ca^2+^] the rate constants of force re-development (*k*_TR_) of d-hESC-CMs toward the same final fractional force level *F*_n_ as in hvMFs (*F*_n_ = *F*_ACT_/*F*_ACT,max_), and plotted *k*_TR_ as function of *F*_n_ (Figures 3A–D). Relationships between *k*_TR_ and *F*_n_ obtained for d-hESC-CMs and hvMFs before and after PKA were fitted using the equation *k*_TR_ = *g*_app_/(1−*F*_n_·*f*_app,max_/(*f*_app,max_+*g*_app_)), where *f*_app,max_ (*f*_app_ at *F*_n_ = 1) and *g*_app_ are the probabilities (rate constants) of cross-bridges to enter and leave the force-generating states, respectively (Brenner, 1991a; Poggesi et al., 2005). Plots of *F*_n_ vs. *k*_TR_ curves are shown with 95% confidence intervals (95%-CI) to compare separately d-hESC-CMs ± PKA (Figure 3A), hvMFs ± PKA (Figure 3B), d-hESC-CMs with hvMFs (Figure 3C), and d-hESC-CMs + PKA with hvMFs + PKA (Figure 3D).

For d-hESC-CMs at force levels below ~33% of *F*_ACT,max_ (and below ~15–20% of *F*_ACT,max_ for hvMFs), it was not possible to reliably measure *k*_TR_, because the laminar solutions flow exerted a hydrodynamic pressure on d-hESC-CMs (and on hvMFs) which perturbed the slow exponential rise of the recorded force signal.

**Figure 4:**
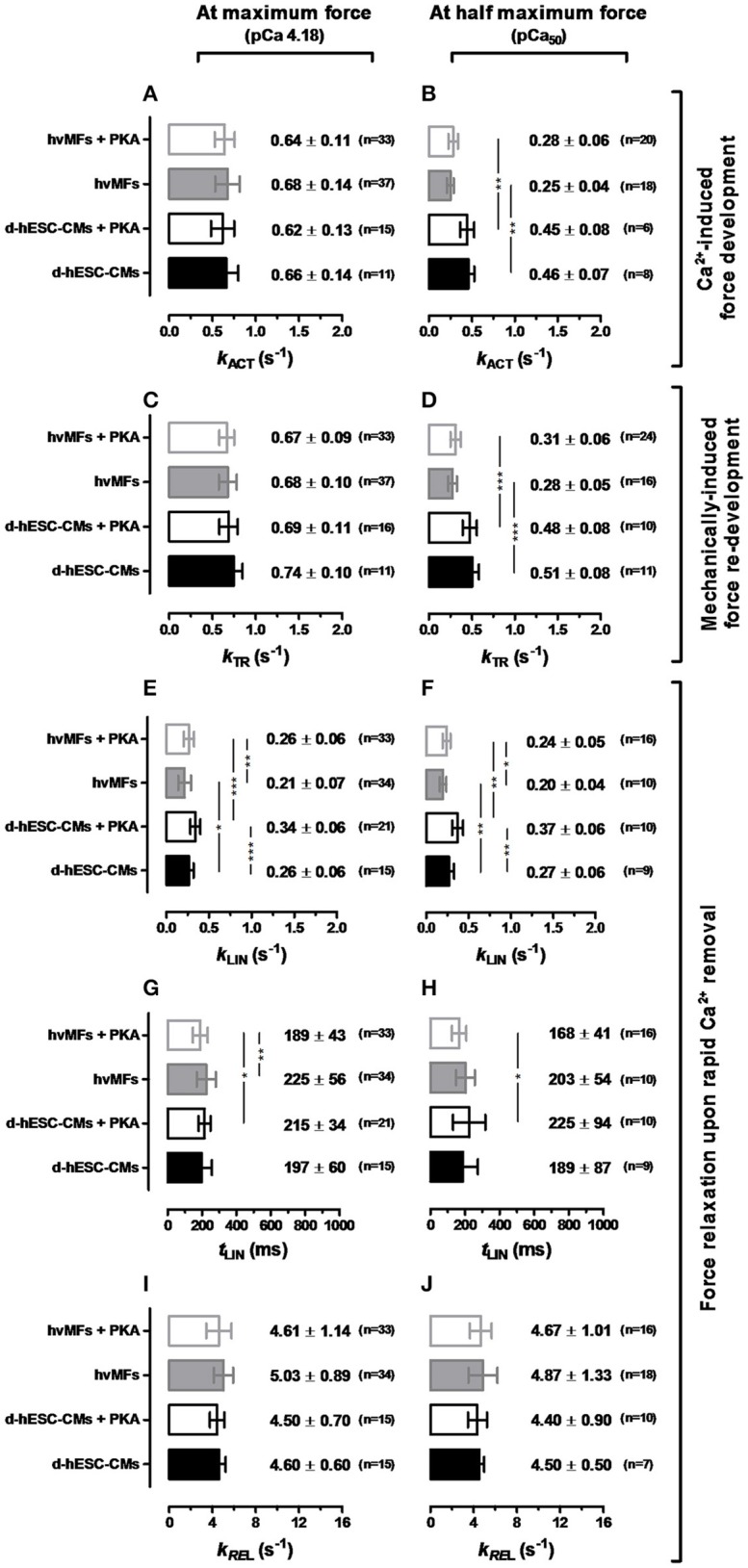
Contractile force kinetic parameters (*k*_ACT_ in **A,B**, *k*_TR_ in **C,D**, *k*_LIN_ in **E,F**, *t*_LIN_ in **G,H**, *k*_REL_ in **I,J**) of d-hESC-CMs (black bars) and hvMFs (gray bars) in the absence (filled bars) or presence (open bars) of PKA-treatment applied prior to measurements. Left panel, at saturating [Ca^2+^] (pCa 4.18). Right panel, at [Ca^2+^] where *F*_ACT_ was half of the maximum force (pCa_50_). Data shown as mean ± *SD*; ^*^p < 0.05, ^**^p < 0.01, ^***^p < 0.001. *n* = 7–21 for d-hESC-CMs; *n* = 10–37 for hvMFs.

We found that for both types of myofibrils receiving or not the PKA-treatment, the *F*_n_ vs. *k*_TR_ curves (Figures 3A,B) were similar and the 95%-CIs were almost completely overlapped along the entire range of recorded fractional force levels (0 < *F*_n_≤1). At pCa_50_ (*F*_n_ = 0.5), PKA had no effect on *k*_ACT_ and *k*_TR_ (Figures 4B,D), as at saturating [Ca^2+^] (*F*_n_ = 1; Figures 4A,C).

We also compared d-hESC-CMs and hvMFs before and after PKA-treatment (Figures 3C,D), respectively. Despite the fact that at saturating [Ca^2+^] *k*_ACT_ and *k*_TR_ values were similar when d-hESC-CMs were compared to hvMFs (Figures 4A,C), at submaximal force levels they were different (Figures 3C,D, 4B,D). 95%-CIs of the *k*_TR_ vs. *F*_n_ curves for d-hESC-CMs and hvMFs did not overlap at intermediate fractional force levels (0 < *F*_n_ < 1) before and after PKA-treatment, respectively (Figures 3C,D).

These results indicate that cross-bridge kinetics, determining the force rise toward submaximal force levels, were different for d-hESC-CMs and hvMFs, while PKA-mediated phosphorylation had no significant effect.

#### Kinetics of force decay during relaxation

Here we determined *k*_LIN_ (the rate constant of the first relaxation phase) which is related to tension cost (= ATPase/Force ~ *g*_app_) (Brenner, 1988; Weber et al., 2016), while the second relaxation phase (described by the rate constant *k*_REL_) is related to the rapid drop of force and cardiomyocyte re-lengthening that contributes to the rapidity of the ventricular pressure decay (i.e., during diastole) (Stehle and Iorga, 2010).

*k*_LIN_ values corresponding to the relaxation from full Ca^2+^-activation and from pCa_50_ to pCa > 8 were compared (Figures 4E,F), revealing that *k*_LIN_ was the same for both activation levels either in d-hESC-CMs or in hvMFs. This suggests that kinetics of cross-bridges leaving the force-generating states are not linked to the Ca^2+^-dependent occupancy of the previous force levels (Stehle et al., 2003). PKA-treatment accelerated the first relaxation phase of both d-hESC-CMs and hvMFs. *k*_LIN_ of d-hESC-CMs treated with PKA had the highest value (Figures 4E,F). Independent of PKA-treatment (± PKA) and of the previous force level (at pCa 4.18 or pCa_50_), *k*_LIN_ for d-hESC-CMs was significantly faster than for hvMFs (Figures 4E,F).

Duration of the first relaxation phase (*t*_LIN_) after rapid Ca^2+^ removal, i.e., the time until the sequential sarcomere lengthening (Videos 2–4) inducing the fast relaxation phase started (Stehle et al., 2002a,b; Poggesi et al., 2005), was similar in d-hESC-CMs and in hvMFs (Figures 4G,H). With PKA-treatment, it became significantly shorter only for hvMFs compared to d-hESC-CMs.

Due to the reduction in strain on the cross-bridges during the second relaxation phase (Figure 1C-right panel, Videos 2–4), the redistribution of cross-bridges toward the non-force-generating states was accelerated by ~10–20 times from the *k*_LIN_ values (<0.4 s^−1^) to the values of *k*_REL_ of ~4–5 s^−1^ for both d-hESC-CMs and hvMFs (Figures 4I,J). Whereas, strained cross-bridges during the first relaxation phase left the force-generating states faster (*p* < 0.05) after PKA-treatment (higher *k*_LIN_), kinetics of the second relaxation phase (*k*_REL_) were not significantly affected by PKA-treatment for both d-hESC-CMs and hvMFs (Figures 4I,J), as it was previously reported for hvMFs (Walker et al., 2011).

### Protein analysis of d-hESC-CMs reveals a sarcomeric protein isoform pattern which is different from hvMFs

The observed differences in myofibrillar force kinetics between d-hESC-CMs and hvMFs suggest a distinct isoform pattern of some sarcomeric proteins between these two contractile systems. Therefore, we have next focused on the identification of the isoforms of the myosin essential light chain (MLC-1), myosin regulatory light chain (MLC-2), myosin binding protein C (MyBP-C), troponin I (TnI), troponin T (TnT) and tropomyosin (Tm) of d-hESC-CMs compared to hvMFs. Since different phosphorylation of some sarcomeric proteins could also contribute to the observed differences in force kinetics, we also investigated sarcomeric protein phosphorylation.

#### Immunofluorescence

##### Myosin heavy chain

**Figure 5:**
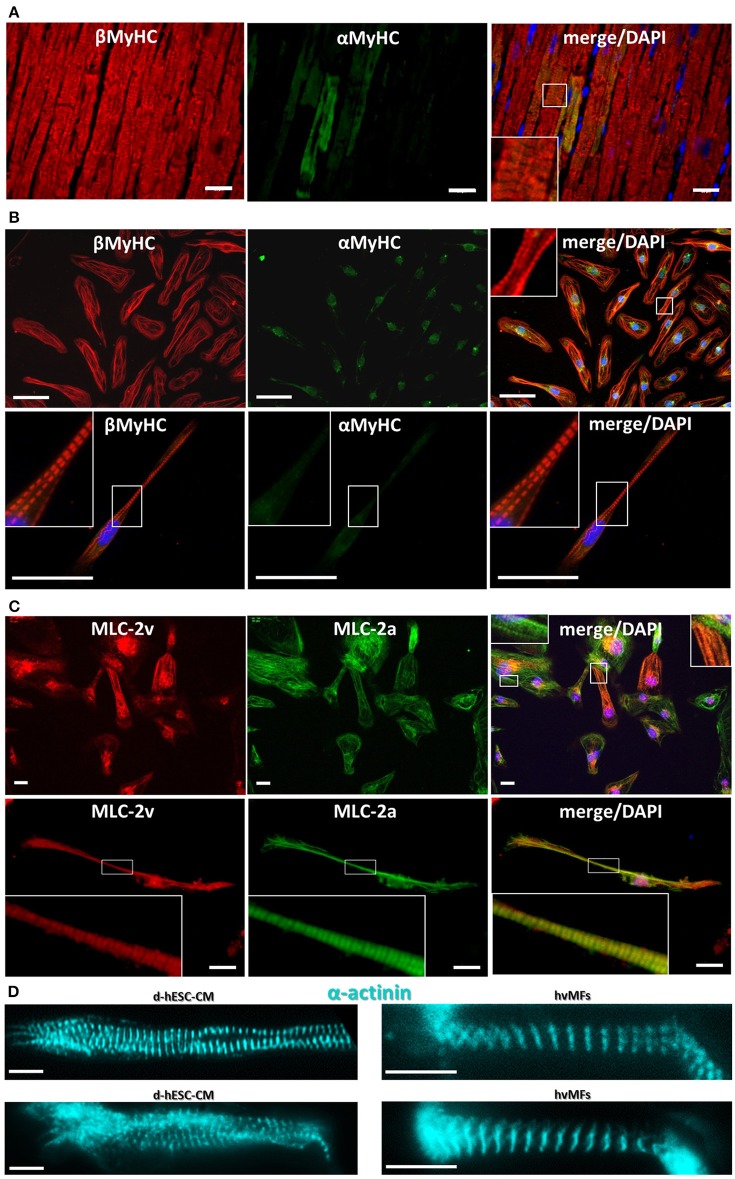
**(A)** Cryosection from human adult ventricular donor tissue immunostained against αMyHC (green fluorescence) and βMyHC (red fluorescence). A few cardiomyocytes show sarcomeres with some αMyHC expressed among the predominant βMyHC isoform. **(B)** Double stained (as in **A**) d-hESC-CMs expressing essentially only βMyHC isoform in their sarcomeres. Some non-sarcomere specific green fluorescence is seen in the αMyHC staining. Images in first row show multiple cells; second row, single cell. **(C)** d-hESC-CMs immunostained against MLC-2v (red) and MLC-2a (green) show a heterogeneous expression of both MLC-2 isoforms. Images in first row show multiple cells; second row, single cell. **(D)** d-hESC-CMs (left) and hvMFs (right) immunostained against α-actinin prior to Ca^2+^-activation in the micromechanical setup. Blue, DAPI for staining of nuclei. Scale bars: 20 μm **(A,C)**, 50 μm **(B)**, 10 μm **(D)**. Insets represent the digital zoom of the selected regions.

Double immunostaining against βMyHC and αMyHC cardiac isoforms of thin slices of human adult ventricular tissue from which hvMFs were isolated revealed only small myofibrillar regions with a mixture of both isoforms, while the rest of sarcomeres were only βMyHC positive. One example with αMyHC-positive CMs is shown in Figure 5A. With long-term (>35 days) cultivation of hESC-CMs on laminin-coated stiff isotropic glass surfaces, MyHC switches to essentially only the βMyHC isoform, as previously described (Weber et al., 2016) and shown here by double immunostaining against both cardiac MyHC isoforms (Figure 5B, Figure S2).

##### Myosin light chain-2

Some d-hESC-CMs plated up to 60 days were immunostained against ventricular (MLC-2v) and atrial (MLC-2a) isoforms of MLC-2 protein (Figure 5C, Figure S3). From 47 analyzed double immunostained d-hESC-CMs (considering only MLC-2a/v incorporated into sarcomeres), ~43% were positive only for MLC-2a, while ~57% of them were positive for both MLC-2a/v isoforms, showing heterogeneous expression of both MLC-2 isoforms in the cells, with a possible predominance of MLC-2a.

Fluorescent immunostaining against MyHC (Figures 5B, Figure S2), MLC-2 (Figures 5C and Figure S3), or α-actinin (Figures 1B, 5D, Video 2) provides information regarding the distribution of myofibrils and sarcomeres within single hESC-CMs compared to adult ventricular tissue (Figure 5A) and single hvMFs-bundles (Figure 5D). Despite morphological (Figures 5B,C, Figures S2, S3) and ultrastructural (Weber et al., 2016) maturation of long-term (>35 days) plated hESC-CMs, myofibrillar density, overall alignment (in particular, the transversal alignment of Z-disks along the myofibrillar bundle) and cardiomyocytes elongation were apparently reduced in single hESC-CMs (Figures 1A_1_,D_3_, 5B–D) than in ventricular CMs (Weber et al., 2016) or within single hvMFs-bundles (Figures 1A_2_, 5A,D).

#### Overview of sarcomeric proteins by gel electrophoresis

**Figure 6:**
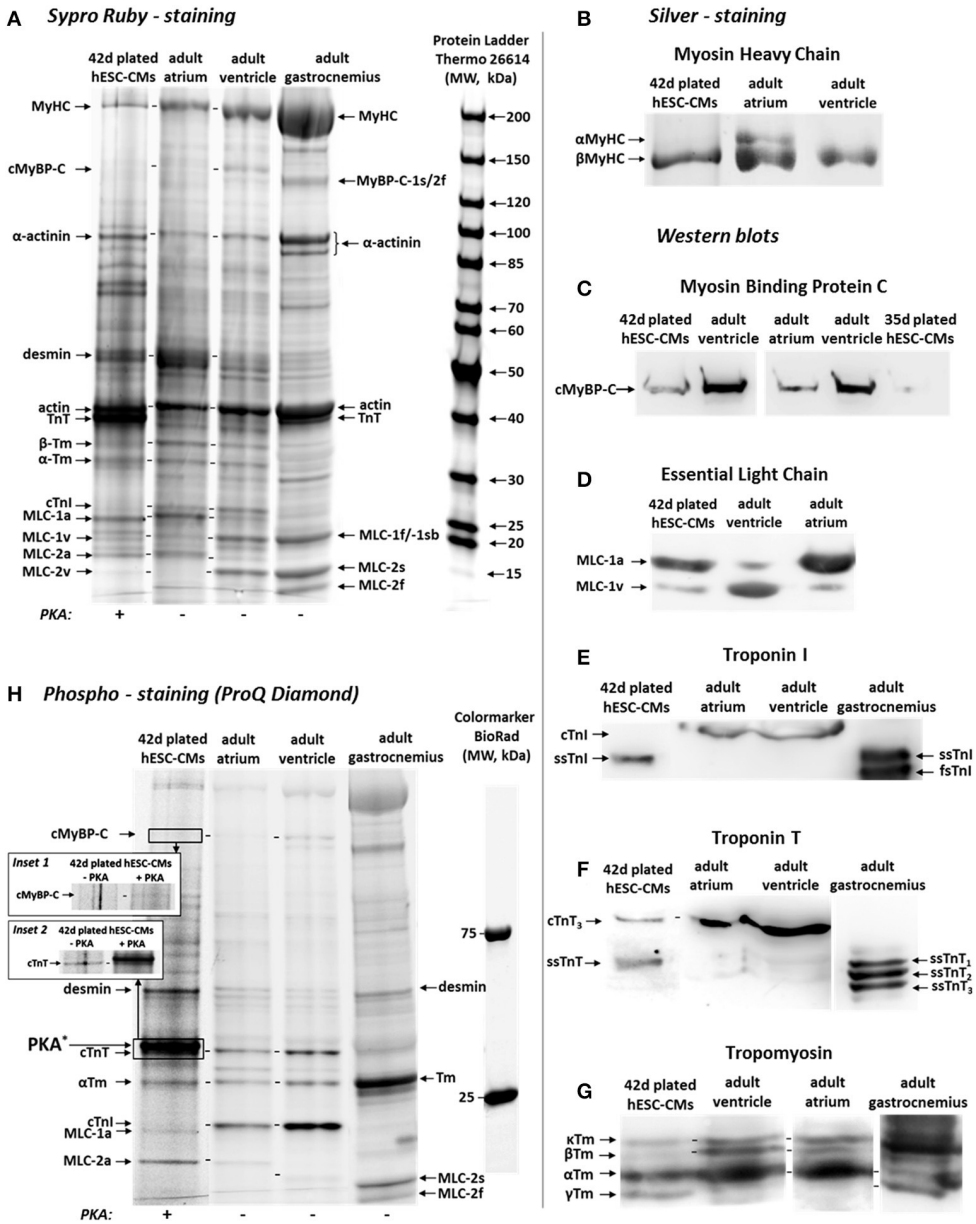
**(A)** Sypro Ruby-stained SDS-gel showing the bands corresponding to different sarcomeric protein isoforms of 42 days plated d-hESC-CMs (lane 1, +PKA), human adult atrial (lane 2, −PKA), ventricular (lane 3, −PKA), and skeletal (*M. gastrocnemius*) muscle (lane 4, −PKA) samples. Donor atrial, ventricular, and gastrocnemius adult muscle samples and the 5th lane loaded with protein ladder were used to identify the sarcomeric protein isoforms observed in d-hESC-CMs. **(B)** Analysis of silver-stained gels revealed an essentially exclusive expression of the βMyHC isoform in both d-hESC-CMs and hvMFs showing not detectable αMyHC isoform. For comparison, demembranated atrial tissue (with αMyHC isoform detected) is shown. **(C–G)** Western Blot analysis showing the presence of the following sarcomeric protein isoforms in d-hESC-CMs: **(C)** cMyBP-C (as detected in adult human ventricular and atrial samples); **(D)** MLC-1a and MLC-1v essential light chains; **(E)** Slow skeletal TnI (ssTnI); note, cardiac TnI (cTnI) was detectable only in adult ventricular and atrial samples; **(F)** Adult cardiac TnT (cTnT_3_) and slow skeletal TnT (ssTnT); **(G)** Tropomyosin isoforms: αTm-, βTm-, κTm-, and γTm-isoforms. **(H)** Phospho-stain (ProQ Diamond) of the gels in **(A)**, showing phosphorylation of some sarcomeric proteins. Insets 1 (cMyBP-C) and 2 (cTnT): d-hESC-CMs treated (+PKA) or not treated (−PKA) with protein kinase A. Lane 1 (+PKA) and inset 2, right lane (+PKA): intense band corresponds to the PKA enzyme itself. Lanes 2, 3, 4: demembranated tissues of adult muscles shown here were not treated with PKA.

In d-hESC-CMs, we could clearly identify the bands of MyHC, α-actinin, desmin, actin, TnT, α and β isoforms of Tm, TnI, atrial (MLC-1a, MLC-2a) and ventricular (MLC-1v, MLC-2v) isoforms of the MLC-1 and MLC-2 proteins (Figure 6A, lane 1), and compared them to those in atrial (lane 2), ventricular (lane 3), and skeletal (lane 4, *M. gastrocnemius*) adult human muscles.

In d-hESC-CMs and in the homogenized ventricular tissue from which hvMFs were prepared for functional investigations, αMyHC isoform was below the detection limit (Figure 6B). MLC-1a (~84%; Figure 6A) and MLC-2a (81.5 ± 2.1%, *n* = 3; Figure 6A, Figure S4D) were the predominant protein isoforms expressed in d-hESC-CMs, similar to the atrial sample (~89% for MLC-1a, ~95% for MLC-2a; Figure 6A), while MLC-2v was 18.2 ± 2.5% (*n* = 3) (Figure 6A, Figure S4D).

#### Western blot analysis

##### Myosin binding protein C

The cardiac isoform of this protein (cMyBP-C) was clearly identified in d-hESC-CMs with its corresponding band at the same position as in ventricular and in atrial samples (Figure 6C, Figure S4A), being slightly above to the position of skeletal MyBP-C-1s/2f isoforms observed in the sample of *M. gastrocnemius* (Figure 6A).

##### Myosin light chain-1

Western blot analysis confirmed the presence of both MLC-1a (86.3 ± 3.3%, *n* = 3) and MLC-1v (13.7 ± 3.3%, *n* = 3) isoforms in d-hESC-CMs (Figure 6D, Figure S4B), but the MLC-1a isoform predominated.

##### Troponin I

The cardiac isoform of TnI (cTnI) was below the detection limit in d-hESC-CMs, while it was the only TnI-isoform in both adult ventricular and atrial samples. Instead, the slow skeletal (ssTnI) isoform was present in d-hESC-CMs, corresponding to the position of the ssTnI isoform identified in the *M. gastrocnemius* (Figure 6E, Figure S4C).

##### Troponin T

In d-hESC-CMs, both cardiac cTnT (~33%) and slow skeletal ssTnT (~67%) isoforms were detected, while in the adult ventricular and atrial samples, only the cTnT isoform was present (Figure 6F).

##### Tropomyosin

For d-hESC-CMs, we observed four bands, as the antibody was specific for all tropomyosin isoforms (Figure 6G). αTm, βTm, and κTm isoforms provided distinct bands, while αTm predominated in all cardiac samples. For d-hESC-CMs, additionally a farther migrating band appeared. This band was less visible in adult cardiac samples and seems to correspond to the last band observed in *M. gastrocnemius* sample, which could be the γTm isoform (Figure 6G; Jagatheesan et al., 2010; Marston et al., 2013).

#### Phosphorylated sarcomeric proteins

Overall, phospho-staining (ProQ Diamond; Figure 6H) revealed that the following sarcomeric proteins were phosphorylated in d-hESC-CMs (Figure 6H, lane 1; + PKA): cMyBP-C (inset 1; ± PKA), desmin, cTnT (inset 2; ± PKA), αTm, MLC-1a, and MLC-2a. For desmin, contributions of cytoskeletal desmin in d-hESC-CMs to the detected band should be considered, because the cytoskeletal network most likely was not completely removed from detergent-treated d-hESC-CMs. Phospho-stained bands corresponding to the phosphorylated cTnT and αTm isoforms were present in all samples [Figure 6H, lane 1 (+PKA), lanes 2,3 (−PKA)]. In adult ventricular and atrial samples, the phosphorylated form of cTnI was clearly visible. Yet, in d-hESC-CMs (±PKA) this band was missing, likely because the cTnI isoform was below detection limit. In d-hESC-CMs, we observed a weak band corresponding to the phosphorylated MLC-1a, which was absent or under the detection limit in adult atrial and ventricular samples (Figure 6H). MLC-2a isoform was also phosphorylated in d-hESC-CMs and more intense than the MLC-1a isoform.

In conclusion, d-hESC-CMs contain sarcomeric protein isoforms found not only in human adult ventricular samples, but also in the atrial and skeletal muscles of the adult human.

## Discussion

In the present study, contractile function of βMyHC isoform-expressing myofibrils within d-hESC-CMs was characterized in comparison to hvMFs. We found that maximum generated force was reduced and Ca^2+^-sensitivity of force was increased, and that kinetics of force re-development at submaximal activation levels and kinetics of the slow relaxation phase were faster in d-hESC-CMs compared to hvMFs. Analysis of sarcomeric protein isoform patterns in both types of myofibrils revealed that d-hESC-CMs express slow skeletal TnI instead of the cTnI isoform normally found in adult ventricles. For other sarcomeric proteins involved in force regulation, non-ventricular isoforms were detected in d-hESC-CMs. The sarcomeric protein isoform pattern of hESC-CMs rather corresponds to cardiomyocytes in the developing human ventricle. The different protein isoforms can explain the differences in functional parameters.

### Developmental aspects related to sarcomeric protein isoforms that modulate contractile function

The βMyHC isoform is predominantly expressed in adult, neonates, and fetal ventricles (Bouvagnet et al., 1987; Miyata et al., 2000; Reiser et al., 2001; Elhamine et al., 2016; Racca et al., 2016). Therefore, it is an indicator for ventricular-like cardiomyocytes in humans during developmental and adult stages. βMyHC-expression can be important for disease modeling in hESC-CMs, e.g., when questions regarding HCM or DCM-related βMyHC-mutations and their functional effects are addressed (Jung and Bernstein, 2014; Pioner et al., 2016).

Both, MLC-1a and MLC-1v isoforms are present during fetal development of the human ventricle (at mid-gestation, ~50% MLC-1a). During the months after birth, MLC-1a is being downregulated in the ventricle to low or even undetectable amounts (Elhamine et al., 2016). In adulthood, MLC-1a remains present only in the atria (Cummins et al., 1980; Cummins and Lambert, 1986). Under chronic hemodynamic overload, MLC-1a becomes re-expressed in the adult ventricular myocardium, affecting contractile properties by forming βMyHC·MLC-1a/v heterodimers (Schaub et al., 1998). In the present study, hESC-CMs cultured on a stiff surface expressed both MLC-1a/v isoforms, but the atrial (fetal) isoform predominated (Figures 6A,D, Figure S4B).

The MLC-2v isoform is predominantly expressed in the human developing fetal (e.g., 18–21 weeks gestational age) and in the adult (young or aged) ventricles, and it remains predominantly expressed even in some diseased ventricles (Cummins et al., 1980; Cummins and Lambert, 1986). Therefore, the MLC-2v isoform was previously used as chamber-specific marker to sort hPSC-CMs (Bizy et al., 2013). Nevertheless, during early embryonic stages of cardiogenesis, both atrial and ventricular isoforms of MLC-2 coexist in sarcomeres, while their genes are differentially regulated (Chien et al., 1993; Vestergaard et al., 2017). For instance, in the embryonic heart tube, MLC-2v isoform predominates only in the part of the cTnI isoform-expressing area (Fijnvandraat et al., 2003). In the hESC-CMs differentiated by Wnt-pathway modulators (Kempf et al., 2014), both MLC-2a/v isoforms were expressed (Figure 6A), as also previously reported for some hPSC-CMs (Bedada et al., 2014; Vestergaard et al., 2017). Yet, in the present study, the atrial isoform predominated in hESC-CMs (Figures 5C, 6A, Figure S4D). Notably, in our previous study, hESC-CMs cultured on a stiff surface (>35 days) that were differentiated using a p38-MAPK inhibitor (Xu et al., 2009; Kempf et al., 2011), expressed predominantly the MLC-2v isoform (Weber et al., 2016). This suggests that specific differentiation and cultivation conditions of hPSC-CMs could influence the transcriptional activity of MLC-1/-2 genes, and that the ventricular isoform expression of MLC-1/-2 is uncoupled from βMyHC-expression. This view is supported by previous findings showing that during the development of human ventricles and atria, mixed isotype assemblies occur *in vivo* (Cummins and Lambert, 1986; Vestergaard et al., 2017). It was suggested that from the pool of various genes related to these proteins, those are preferably expressed during development which build heteromeric myosin assemblies with functional advantages optimally adapted to a specific contractile task (Schaub et al., 1998).

For cMyBP-C no change in isoform expression has been observed during development of cardiomyocytes (i.e., lack of transcomplementation) (Gautel et al., 1998). In our work, western blot analysis of d-hESC-CMs confirmed the presence of cMyBP-C (Figure 6C, Figure S4A).

Regarding cTnT, four cardiac isoforms (cTnT_1−4_) can be expressed in human cardiomyocytes through combinatorial alternative mRNA splicing in a developmentally regulated manner (Gomes et al., 2002). cTnT_1,2,4_ and ssTnT isoforms are usually expressed in early fetal heart (Barton et al., 2004). In the adult heart, only cTnT_3_ is present and ssTnT might be expressed at low levels, as response to myocardial stress. In this study, hESC-CMs express both adult cTnT_3_ and ssTnT isoforms (Figure 6F), while the other fetal cTnT_1_ and cTnT_2_ isoforms, previously reported in early stages of fetal ventricles (Racca et al., 2016), were not detected. In another study with hPSC-CMs, only one cTnT isoform corresponding to the 130 days gestational stage of the human fetal ventricle was reported (Pioner et al., 2016).

TnT has several isoforms and different genes control its expression in cardiac, slow- and fast-twitch skeletal muscles. TnT isoform genes are connected in chromosomal DNA to TnI isoform genes, e.g., the cTnT-gene is paired with the ssTnI-gene and the ssTnT-gene is paired with cTnI-gene, respectively (Wei and Jin, 2011). Such pairing reflects original functional linkages, because in embryonic cardiac muscles the cTnT-gene is expressed together with the ssTnI-gene (Gomes et al., 2002; Wei and Jin, 2011). Further developmental transition of cTnT and cTnI isoforms seems to be regulated by different mechanisms in mammals (Gao et al., 1995). Interestingly, the ssTnT-to-cTnT isoform shift occurs earlier than the ssTnI-to-cTnI shift (Siedner et al., 2003; Elhamine et al., 2016), which may explain the TnI- and TnT-isoform patterns of the d-hESC-CMs observed here (Figures 6E,F, Figure S4C).

In the adult healthy human ventricle, essentially only cTnI is found. Fetal ventricles express both cTnI and ssTnI, which are regulated at the level of gene transcription. ssTnI is progressively downregulated within the first year after birth, similar to the fetal MLC-1a isoform (Elhamine et al., 2016; Racca et al., 2016). In the d-hESC-CMs analyzed here only ssTnI was detected (Figure 6E, Figure S4C), while in other studies with hPSC-CMs, both ssTnI and cTnI isoforms were reported (Bedada et al., 2014; Pioner et al., 2016).

Previous studies indicated that the thyroid growth hormone (T3) increased the cTnI/ssTnI ratio in developing cultured cardiomyocytes (Riedel et al., 2005), promoting also different other maturation aspects of hPSC-CMs (Yang et al., 2014b). However, T3 also causes a shift from the slow βMyHC to the fast αMyHC isoform in hESC-CMs, enhancing their sarcomeric force kinetics (Weber et al., 2016). Therefore, further studies are needed to clarify how cTnT/ssTnT and cTnI/ssTnI ratios can be increased in hESC-CMs while maintaining the predominant expression of βMyHC. Raising cTnT/ssTnT ratio in hPSC-CMs might be particularly important, because the cTnT isoform is commonly used as a cardiac lineage differentiation marker during cells selection procedure. However, to our knowledge, this criterion does not exclude selection of cardiomyocytes expressing both cTnT and ssTnT isoforms and it does not distinguish between the adult cTnT_3_ and the other fetal cardiac isoforms of TnT.

The αTm and βTm isoforms are both expressed in developing and adult heart. Although the expression of the βTm isoform increases somewhat in transition from fetus to adult, αTm remains the predominant isoform (Muthuchamy et al., 1993; Rajan et al., 2010; Marston et al., 2013). The κTm isoform results from alternative splicing of the *TPM1*-gene encoding for αTm and it is expressed only at low protein levels (Rajan et al., 2010). The γTm isoform seems to be less expressed or even absent in adult human ventricles, while it is usually found in slow-twitch skeletal muscles (Jagatheesan et al., 2010; Rajan et al., 2010; Marston et al., 2013). In this study, all four α, β, κ, and γ isoforms of Tm were detected in d-hESC-CMs and the band of γTm was even stronger than that of either βTm or κTm isoform (Figure 6G). Most importantly, in d-hESC-CMs the αTm isoform was predominant, as in the adult ventricular sample (Figure 6G).

In the present study, hESC-CMs cultivated on coverslips exhibited a sarcomeric protein isoform pattern similar to cardiomyocytes of the early fetal stage or even embryonic stage of the developing human ventricle. The deviation from the adult ventricular sarcomeric protein isoform pattern of d-hESC-CMs likely affected contractile function of their myofibrils, as discussed in the following section.

### Differences in contractile function between d-hESC-CMs and hvMFs are related to protein isoform expression

#### Passive force

The significantly lower passive force (*F*_pass_) for d-hESC-CMs vs. hvMFs (Figure S1), could be due to the presence of the more compliant and longer fetal-N2BA titin isoform (Kruger and Linke, 2009). This is supported by other studies which showed that the fetal-N2BA isoform predominated in hiPSC-CMs (Hinson et al., 2015). *F*_pass_ for hvMFs was slightly, but significantly (*p* < 0.05) diminished following PKA-treatment, while for d-hESC-CMs, PKA had no effect (Figure S1). It was previously shown that adult donor ventricular tissue contains the shorter compliant N2BA and the stiffer N2B titin isoforms in the ratio N2BA:N2B~0.5 and only N2B is target for PKA-mediated phosphorylation (Kruger and Linke, 2009). To determine *F*_ACT_ we subtracted *F*_pass_ from the recorded active force.

#### Contractile function at saturating [Ca^2+^]

We found that maximum isometric force was significantly smaller for d-hESC-CMs (~42 kPa) than for hvMFs (~94 kPa), presumably due to less well-aligned myofibrils within d-hESC-CMs compared to more compact hvMFs-bundles (e.g., Figures 1A, 5D), but differences in sarcomeric protein isoform composition (Figure 6, Figure S4) could also contribute to the lower isometric force.

Upon sudden increase of [Ca^2+^] from relaxation (>pCa 8) to pCa 4.18, force raised mono-exponentially without lag (Figure 1C) and with very similar *k*_ACT_ in both d-hESC-CMs and hvMFs. This suggests that myofibrils within d-hESC-CMs, as previously shown for hvMFs (Stehle et al., 2002b; Poggesi et al., 2005), rapidly equilibrate with the surrounding Ca^2+^-defined solution and that the measured kinetics are not rate-limited by diffusional events. Because *k*_ACT_ in both types of myofibrils is similar to *k*_TR_, kinetics of the force rise upon Ca^2+^-activation of d-hESC-CMs, as shown before for hvMFs (Stehle et al., 2002b), seem to be primarily governed by cycling cross-bridges rather than by the much faster Ca^2+^-controlled switch on-and-off of the regulatory proteins of the thin filaments. *k*_TR_ for d-hESC-CMs and hvMFs was very similar to *k*_TR_ previously determined for hvMFs (Stehle et al., 2002b; Piroddi et al., 2007). This suggests that cross-bridge cycling kinetics at full calcium-activation seems not to be affected by the observed differences in thin filaments regulatory protein- and myosin light chain-isoforms.

#### Contractile function at intermediate [Ca^2+^]

Cardiomyocytes in the living heart and in cell culture are operating at submaximal intracellular [Ca^2+^]. At intermediate [Ca^2+^], development-dependent isoform differences of some sarcomeric proteins may influence the steady-state force and cross-bridge cycling kinetics (Metzger et al., 2003; Siedner et al., 2003; Racca et al., 2016).

Ventricular myofibrils and cardiomyocytes expressing the ssTnI isoform exhibit an increased Ca^2+^-sensitivity of force compared to those containing cTnI, and even a partial expression of ssTnI, co-expressed with cTnI, seemed to have a dominant Ca^2+^-sensitizing effect (Metzger et al., 1994, 2003; Siedner et al., 2003; Elhamine et al., 2016; Racca et al., 2016). Therefore, it is very likely that the ssTnI, solely detected in the d-hESC-CMs, contributes to the observed higher Ca^2+^-sensitivity of force when compared to adult hvMFs (Figures 2A,C) containing only cTnI (Figure 6E, Figure S4C). In addition, cTnI was phosphorylated in hvMFs (Figure 6H), which reduces Ca^2+^-sensitivity and thus, this may add to the large difference in Ca^2+^-sensitivity of force between d-hESC-CMs and hvMFs (ΔpCa_50_ = +0.24) (Figures 2A,B). ssTnI does not have the PKA-dependent phosphorylatable N-terminal extension of cTnI. Therefore, effects of PKA-treatment of d-hESC-CMs on pCa_50_ cannot be mediated through TnI phosphorylation.

In contrast to the Ca^2+^-sensitizing effect of ssTnI, the ssTnT isoform, detected together with cTnT in d-hESC-CMs (Figure 6F), was shown to reduce the Ca^2+^-sensitivity of force in cardiac fibers (Pinto et al., 2012). However, the effect of ssTnT apparently does not dominate the Ca^2+^-sensitivity of force for d-hESC-CMs compared to hvMFs.

αTm is the predominant tropomyosin isoform expressed in both d-hESC-CMs and hvMFs. βTm and κTm isoforms were shown to exert increasing and decreasing effects on Ca^2+^-sensitivity of force, respectively (Jagatheesan et al., 2010; Rajan et al., 2010). Therefore, the effects may be compensatory in both, d-hESC-CMs and hvMFs. d-hESC-CMs seem to express additionally the γTm isoform (Figure 6G). Co-expression of γTm in cardiac muscle decreases Ca^2+^-sensitivity of force, and importantly, γTm apparently has a functional dominance over the other Tm isoforms in the regulation of striated muscle performance (Pieples et al., 2002; Jagatheesan et al., 2010). Therefore, the presence of γTm in d-hESC-CMs would argue for a decrease of Ca^2+^-sensitivity of force compared to hvMFs.

Isoform differences of TnI, TnT and Tm observed in d-hESC-CMs vs. hvMFs may affect the fast switch-on/-off equilibrium of the thin filament, determining in d-hESC-CMs a particular thin filament status which might be different from that in hvMFs. γTm is more negatively charged and less flexible than the αTm (Jagatheesan et al., 2010) and, together with the ssTnT/cTnT and ssTnI isoforms in d-hESC-CMs, may alter dynamics of the thin filaments to expose their strong binding sites to myosin heads and to the other actin-interacting partners, e.g., N-terminus of MLC-1 (Rarick et al., 1996) and of cMyBP-C (Craig et al., 2014). All these may provide a feedback to the dynamical status of thin filaments, especially at lower [Ca^2+^], as previously proposed considering the cross-bridge-mediated cooperative activation of thin filaments to further recruit new cross-bridges (Campbell, 1997).

MLC-1 interacts in an isoform dependent manner with the lever arm of MyHC (via non-covalent interactions) and with actin (via electrostatic interactions), providing a possible tether for the myosin head between thick and thin filaments. MLC-1a has a higher affinity for the MyHC lever arm and a lower affinity for actin than the MLC-1v isoform (Morano and Haase, 1997; Hernandez et al., 2007). Therefore, the MyHC·MLC-1a may leave the force-generating states faster than MyHC·MLC-1v. It was shown that a higher MLC-1a content by ~20% in human ventricular fibers increases Ca^2+^-sensitivity of force (pCa_50_) by +0.36 pCa-units (Morano and Haase, 1997; Schaub et al., 1998; Hernandez et al., 2007). MLC-1 can be phosphorylated (Arrell et al., 2001), and this is believed to increase cross-bridge detachment rates, thus shifting the equilibrium of cross-bridges more toward the non-force-generating states, i.e., increasing *g*_app_. Altogether, a predominant expression of MLC-1a in d-hESC-CMs compared to hvMFs (Figures 6A,D, Figure S4B), which seems to be partially phosphorylated (Figure 6H), may contribute to the higher Ca^2+^-sensitivity of force (Figure 2A) and to a distinct modulation of cross-bridge cycling kinetics at submaximal force levels. This is supported here by significantly faster *k*_LIN_ (Figures 4E,F) and *k*_TR_ (Table 2, Figures 3C, 4D) at pCa_50_ for d-hESC-CMs compared to hvMFs.

**Table 2:** Measured force kinetic parameter *k*_TR_ determined at pCa_50_ (*F*_n_ = 0.5) and at a given [Ca^2+^] of pCa 5.80 and the fractional force *F*_n_ (*F*_n_ = *F*_ACT_/*F*_ACT,max_) at pCa 5.80 for d-hESC-CMs and hvMFs, which were either treated (+PKA) or not (−PKA) with protein kinase A.

**Force parameters**	**d-hESC-CMs**		**hvMFs**	
	**−PKA**	**+PKA**	**−PKA**	**+PKA**
*k*_TR_ (s^−1^) at pCa_50_	0.51 ± 0.08*^a^*	0.48 ± 0.08*^b^*	0.28 ± 0.05*^a^*	0.31 ± 0.06*^b^*
	*ns* (*p* = 0.36)		*ns* (*p* = 0.05)	
*k*_TR_ (s^−1^) at pCa 5.80	0.58 ± 0.11^c^	0.46 ± 0.11^d^	0.27 ± 0.06^c^	0.26 ± 0.06^d^
	^*^(*p* < 0.05)		*ns* (*p* = 0.49)	
*F*_n_ at pCa 5.80	0.75 ± 0.06	0.49 ± 0.08	0.50 ± 0.05	0.35 ± 0.10
	^**^(*p* < 0.001)		^**^(*p* < 0.001)	

*Data shown as mean ± SD*.

a−d indicates significant differences for the mean of k_TR_ values between d-hESC-CMs (±PKA) and hvMFs (±PKA); (p < 0.0001, Student's unpaired t-test). p-values are indicated for other comparisons; ns, not significant;

* p < 0.05 and

** *p < 0.001, significant*.

Phosphorylation of MLC-2 was shown to increase *k*_TR_ and Ca^2+^-sensitivity of force (van der Velden et al., 2003a,b). In d-hESC-CMs, both MLC-2a/v isoforms co-exist, with MLC-2a being predominant (Figure 6A, Figure S4D) and phosphorylated (Figure 6H). This may contribute to the higher pCa_50_ (Figure 2) and faster *k*_TR_ at pCa_50_ (Table 2, Figures 3C, 4D) or at a given [Ca^2+^] (e.g., at pCa 5.80 shown in Table 2) for d-hESC-CMs compared to hvMFs.

Altogether, in d-hESC-CMs, functional effects of MLC-1a and ssTnI (both increasing pCa_50_) seem to dominate the effects of ssTnT and γTm (both decreasing pCa_50_) at submaximal forces (Table 2), because d-hESC-CMs had a higher Ca^2+^-sensitivity of force compared to hvMFs, when both were either treated (ΔpCa_50_ = +0.11) or not treated (ΔpCa_50_ = +0.24) with PKA, respectively (Figures 2A,B). However, possible differences between the phosphorylation status of other sarcomeric proteins than cTnI of d-hESC-CMs compared to hvMFs may also affect Ca^2+^-sensitivity.

cMyBP-C is a target for PKA and it is expressed in both types of myofibrils studied here. Even though phospho-staining analysis showed a weak signal for phosphorylation of cMyBP-C (Figure 6H, inset 1), the force-pCa relation of d-hESC-CMs was clearly shifted to higher [Ca^2+^] upon PKA-treatment (ΔpCa_50_ = −0.26; Figure 2C). It would be possible that a change in cMyBP-C phosphorylation contributes significantly to this right-shift of the force-pCa relation, because cTnI was undetected in d-hESC-CMs. It was previously shown that in cardiomyocytes lacking cTnI, the phosphorylation of cMyBP-C is able to decrease Ca^2+^-sensitivity of force (Chen et al., 2010). PKA-treatment reduced less, but significantly Ca^2+^-sensitivity of force in hvMFs vs. d-hESC-CMs (Figure 2C), suggesting that hvMFs were already more phosphorylated than d-hESC-CMs.

**Figure 7:**
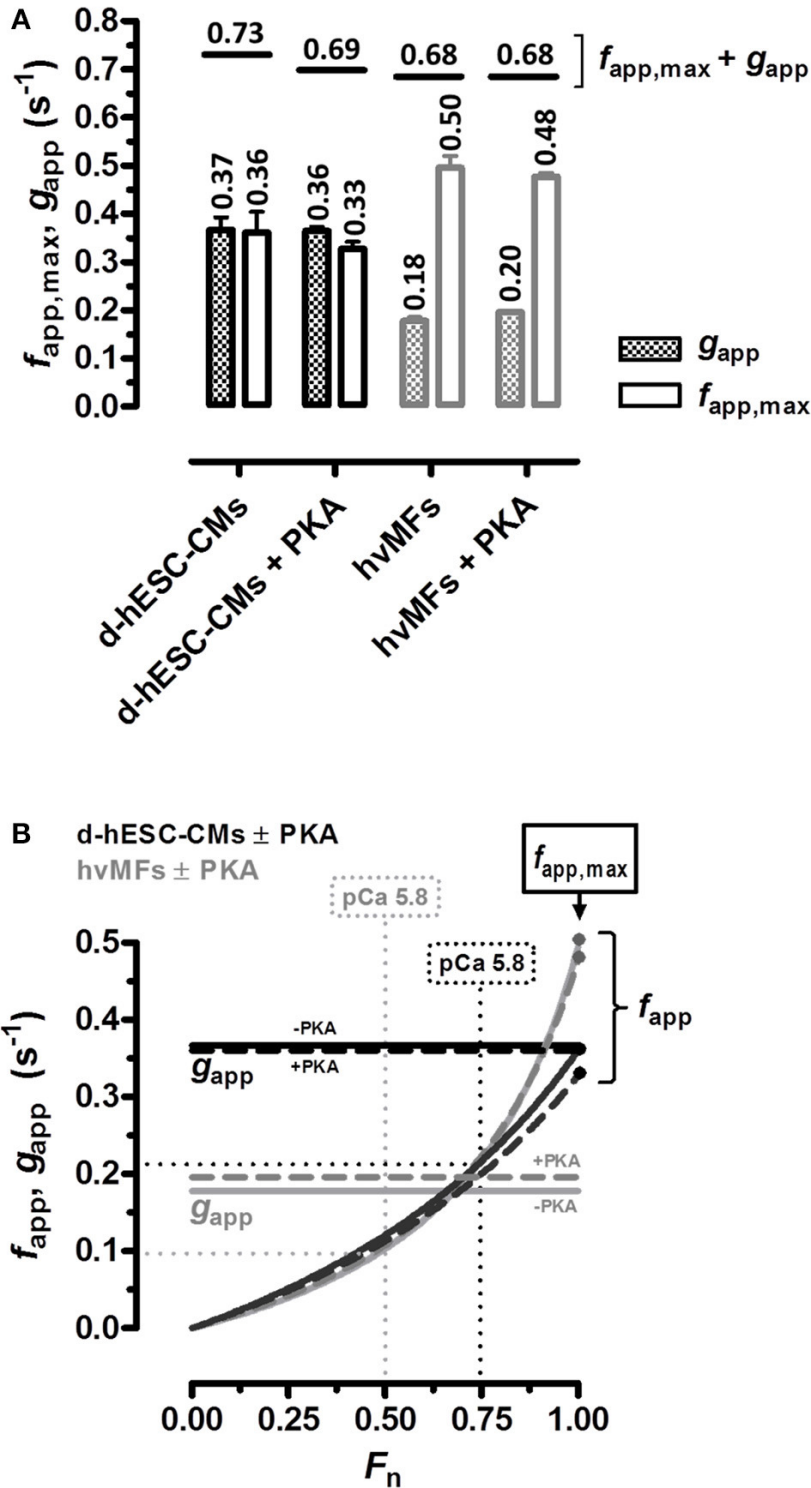
**(A)** *k*_TR_ vs. *F*_n_ data (plotted in Figure 3) was fitted to the equation given in the legend of Figure 3 and yielded the cross-bridge kinetics-related predicted parameters *g*_app_ (patterned bars) and *f*_app,max_ at *F*_n_ = 1 (open bars) for d-hESC-CMs (black bars) and for hvMFs (gray bars) which were either treated or not with PKA. Horizontal short lines correspond to the sum *f*_app,max_ + *g*_app_ predicting *k*_TR_ at saturating [Ca^2+^] (pCa 4.18). Numbers, mean values. Error bars, standard errors (SE). **(B)** From all four curves (black, d-hESC-CMs; gray, hvMFs; solid, −PKA; dashed, +PKA) fitting *k*_TR_ vs. *F*_n_ data shown in Figure 3, *f*_app_ was calculated by subtracting the predicted *g*_app_ values given in A and then plotted against *F*_n_ (0 < *F*_n_ ≤ 1). *g*_app_ is Ca^2+^-independent (horizontal lines); *f*_app_ was positive, monotonically increasing from zero (*F*_n_ = 0) to *f*_app,max_ (*F*_n_ = 1) (Brenner, 1988).

For both d-hESC-CMs and hvMFs at any given submaximal fractional force level (0 < *F*_n_ < 1, *F*_n_ = *F*_ACT_/*F*_ACT,max_), PKA-treatment had no significant effect on measured *k*_TR_ (Figures 3A,B, 4D) and thus, not on *f*_app_+*g*_app_ (= *k*_TR_). *f*_app_ (which depends on [Ca^2+^] modulating the thin filament status) and *g*_app_ (which is independent of [Ca^2+^]) are the rate constants (probabilities) of cross-bridges entering and leaving the force-generating states, respectively (Brenner, 1988). At the same given [Ca^2+^], small differences could eventually exist between *k*_TR_ values determined before and after PKA-treatment, because *f*_app_ is Ca^2+^-sensitive and PKA-treatment decreased Ca^2+^-sensitivity of force. For example, at pCa_50_ (*F*_n_ = 0.5) or at a given [Ca^2+^] (pCa 5.80), *k*_TR_ values are shown in Table 2. It was previously reported with adult hvMFs, involving the same investigation method to determine *k*_TR_ as in the present study, that a shift in Ca^2+^-sensitivity of force following the PKA-treatment did not significantly affect *k*_TR_ at different fractional force levels (Walker et al., 2011). Using the same methodological approach with human donor cardiomyocytes, *k*_TR_ at different [Ca^2+^] was not significantly affected by PKA-treatment (van Dijk et al., 2014). In studies with murine ventricular skinned myocardium, which may have lower endogenous phosphorylation levels than human donor samples, PKA-treatment accelerated *k*_TR_ and this was attributed mainly to cMyBP-C phosphorylation (Stelzer et al., 2006). It was suggested that the acceleration of *k*_TR_ occurred by increasing the rate constants of both cross-bridges detachment and recruitment (Stelzer et al., 2006). In d-hESC-CMs expressing cMyBP-C and lacking cTnI, PKA-treatment enhanced the probability of strained cross-bridges to leave the force-generating states, as the differences (*p* < 0.01) in *k*_LIN_ for d-hESC-CMs ± PKA suggest (Figures 4E,F), but it had no significant impact on less strained cross-bridges, as *k*_REL_ values were similar (Figures 4I,J). *k*_LIN_ was accelerated by PKA-treatment also in hvMFs, but apparently less than in d-hESC-CMs (Figures 4E,F). Therefore, during mechanically loaded cross-bridge turnover at submaximal *F*_n_, the *k*_TR_, which was essentially unaffected by PKA-treatment in d-hESC-CMs (Figures 3A, 4D), would likely result from a relatively small increase of *g*_app_ and decrease of *f*_app_ (to keep *f*_app_+*g*_app_ = *k*_TR_ relatively constant). This could be due to a subtle alteration of thin filaments turning-on/off dynamics in the context of different protein isoform pattern (e.g., lack of cTnI) in d-hESC-CMs compared to hvMFs. However, the potential effects caused by PKA-treatment on *g*_app_ (increasing) and *f*_app_ (decreasing) could not be accurately predicted (large 95%-CI) from *k*_TR_ vs. *F*_n_ dependencies determined at submaximal force levels for d-hESC-CMs (Figure 3A), but such predictions can be used to compare d-hESC-CMs with hvMFs (see below Figures 7A,B).

#### Why maximum isometric force and the rate constant of force re-development at submaximal [ca^2+^] might be different for d-hESC-CMs compared to hvMFs?

From the curves (Figure 3) fitting the *k*_TR_ vs. *F*_n_ data, the apparent rate constants *g*_app_ at *F*_n_ = 0 and *f*_app,max_ at *F*_n_ = 1 can be predicted (Figure 7A) (Brenner, 1988). In d-hESC-CMs, *f*_app,max_ and *g*_app_ are essentially equal, different from the hvMFs where we calculated 2 to 3-fold larger values for *f*_app,max_ compared to *g*_app_ (Figure 7A). Such a difference between *f*_app,max_ and *g*_app_, and even larger ones, were also previously shown for hvMFs (Stehle et al., 2002b; Poggesi et al., 2005; Piroddi et al., 2007). Based on these calculations, the decrease in *F*_ACT,max_ generated by d-hESC-CMs (~42 kPa) vs. hvMFs (~94 kPa), partly results from a decreased occupancy of force-generating cross-bridge states determined by a reduced duty-ratio *f*_app,max_/(*f*_app,max_+*g*_app_) (Brenner, 1988), because *f*_app,max_ is lower and *g*_app_ is higher in d-hESC-CMs compared to corresponding parameters in hvMFs. Yet, if *F*_ACT,max_ is estimated considering only the duty-ratio from the predicted values of *f*_app,max_ and *g*_app_ (Figure 7A), *F*_ACT,max_ for d-hESC-CMs would be ~67% of *F*_ACT,max_ for hvMFs, while the measured force of d-hESC-CMs (42 kPa) was ~45% of the force generated by hvMFs (94 kPa). Therefore, other factors may contribute to the estimated differences, for instance, a less ordered and less compact sarcomere alignment within d-hESC-CMs than that observed in hvMFs-bundles (Figures 1A, 5D) and in healthy cardiomyocytes (Kraft et al., 2013; van Dijk et al., 2014). In developing hESC-CMs, structural maturation of sarcomeres could play an important role for force generation, as previously described in human fetal cardiac muscles (Racca et al., 2016).

While at full Ca^2+^-activation *k*_TR_ is similar for both contractile systems, at submaximal force levels (0 < *F*_n_ < 1), *k*_TR_ was significantly faster for d-hESC-CMs than for hvMFs (Figures 3C, 4D). This is mostly due to a higher *g*_app_ in d-hESC-CMs than in hvMFs (Figure 7A). Higher *g*_app_ of d-hESC-CMs vs. hvMFs may determine an increase of the tension cost (= ATPase/force) that is proportional to *g*_app_ (Brenner, 1988). At lower [Ca^2+^], *f*_app_/*g*_app_ may modulate contractile function in addition to modulation through changes within the regulatory proteins (Brenner, 1988). Predicted curves fitting *k*_TR_ vs. *F*_n_ data (Figure 3C) and *g*_app_ parameters (Figure 7A) were further used to calculate *f*_app_ and then *f*_app_ was plotted against *F*_n_ (Figure 7B). Independent on *g*_app_, *f*_app_ decreases monotonically with decreasing *F*_n_ (Figure 7B). Even if predicted *f*_app_ vs. *F*_n_ curves seem to be similar below *F*_n_~0.75 for both contractile systems (Figure 7B), each fractional force level *F*_n_ is reached at lower [Ca^2+^] in d-hESC-CMs than in hvMFs, because d-hESC-CMs are more Ca^2+^-sensitive than hvMFs (Figure 2). For example, at a given [Ca^2+^] of pCa 5.8, d-hESC-CMs generated 75% of maximum force *F*_ACT,max_ (Table 2) and predicted *f*_app_ = 0.22 s^−1^ (Figure 7B), while hvMFs generated 50% of their maximum force *F*_ACT,max_ (Table 2) and predicted *f*_app_ = 0.10 s^−1^ (Figure 7B). Independent on [Ca^2+^], predicted *g*_app_ was 0.37 s^−1^ for d-hESC-CMs and 0.18 s^−1^ for hvMFs (Figures 7A,B). Therefore, *k*_TR_ (= *f*_app_+*g*_app_) is larger for d-hESC-CMs (0.59 s^−1^) than for hvMFs (0.28 s^−1^) at pCa 5.8.

The aspects discussed here resulted solely from the comparison of hESC-CMs [differentiated and cultivated as previously described (Kempf et al., 2014; Weber et al., 2016)] with hvMFs (isolated from adult human ventricles). Variability in the outcomes cannot be excluded if d-hPSC-CMs would be used which were differentiated in another way and subjected to different maturation conditions.

## Conclusions

In the present study, myofibrillar contractile function was linked to sarcomeric protein isoform pattern of adult hvMFs and of hESC-CMs after several weeks of cultivation on a stiff surface (Weber et al., 2016). Our present results suggest that more mature morphological and ultrastructural aspects (Weber et al., 2016) of hESC-CMs may not necessarily correspond to an overall adult ventricular-like sarcomeric protein isoform pattern and contractile function at the myofibrillar level (Pioner et al., 2016; Racca et al., 2016). Therefore, one might consider not only cTnI (Bedada et al., 2014; Pioner et al., 2016) or MLC-2v (Bizy et al., 2013; Vestergaard et al., 2017) as sarcomeric protein markers for hPSC-CMs differentiated *in vitro*, but also the adult ventricle-specific protein isoforms βMyHC, MLC-1v and cTnT_3_. It would be of great interest to identify the appropriate chemo-mechanical factors to be applied to *in vitro* differentiated hPSC-CMs to speed-up shifting the expression of MLC-1, MLC-2, TnT, and TnI proteins toward the full spectrum of adult ventricular-like sarcomeric protein isoforms in βMyHC expressing hPSC-CMs in a cost-effective manner. On the other hand, *in vitro* differentiated cardiomyocytes with sarcomeric protein isoform composition closer to early stages of the developing human heart, may well provide a model to study e.g., basic mechanistic aspects of contraction in developing human cardiomyocytes, mechanisms of heart failure onset during *in utero* heart development, or inherited cardiac diseases affecting contractile function.

## Author contributions

BI, project design, establishing the micromechanical setup, micromechanical experiments, and functional data analysis and modeling with hESC-CMs and hvMFs, manuscript concept design and manuscript writing; KS, generation, differentiation, and purification of hESC-CMs; NW, MW, and SG, cultivation of hESC-CMs, immunostaining, data analysis, and interpretation; BP, gel-electrophoretic analysis of sarcomeric proteins; CdR, substantial contributions to conception of the project, acquisition of data, and human samples preparation; UM, RZ, TK, and BB, supervising and feedback on the project, intellectual improvement of the manuscript content and structure, manuscript writing. All authors approved the final version of the manuscript.

### Conflict of interest statement

The authors declare that the research was conducted in the absence of any commercial or financial relationships that could be construed as a potential conflict of interest. The reviewer RM declared a past co-authorship with one of the authors CdR to the handling Editor.

## Abbreviations

95%-CI(s): 95% Confidence interval(s)
BDM, 2: 3-Butanedione monoxime
BF: Bright field
[Ca^2+^]: Calcium concentration(s)
CM(s): Cardiomyocyte(s)
CSA: Cross section area
DCM: Dilated cardiomyopathy
DTT: Dithiothreitol
HCM: Hypertrophic cardiomyopathy
FL: Fluorescence
d-hESC-CM(s): demembranated human embryonic stem cell-derived cardiomyocyte(s)
hESC-CM(s): human embryonic stem cell-derived cardiomyocyte(s)
hPSC-CM(s): human pluripotent stem cell-derived cardiomyocyte(s)
hvMFs: human ventricular myofibrils
PhC: Phase contrast
PIC: Protease inhibitor cocktail
PKA: Protein kinase A
SL: Sarcomere length (SL_0_: SL of slack myofibrils).
