# Supplementary material for: Differences in Contractile Function of Myofibrils within Human Embryonic Stem Cell-Derived Cardiomyocytes vs. Adult Ventricular Myofibrils Are Related to Distinct Sarcomeric Protein Isoforms
Source: Front Physiol. 2018 Jan 19;8:1111. doi: 10.3389/fphys.2017.01111 (PMC5780405; doi:10.3389/fphys.2017.01111)
Supplement: Supplementary file 6 [file DataSheet1.pdf]

SUPPLEMENTARY MATERIAL

Differences in contractile function of myofibrils within human embryonic stem cell-derived cardiomyocytes *vs.* adult ventricular myofibrils are related to distinct sarcomeric protein isoforms

Bogdan Iorga[1,2], Kristin Schwanke[3], Natalie Weber[1], Meike Wendland[1], Stephan Greten[1], Birgit Piep[1], Cristobal G. dos Remedios[4], Ulrich Martin[3], Robert Zweigerdt[3], Theresia Kraft[1], Bernhard Brenner[1]

[1] Department of Molecular and Cell Physiology, Hannover Medical School, Hannover, Germany

[2] Department of Physical Chemistry, Faculty of Chemistry, University of Bucharest, Bucharest, Romania

[3] Department of Cardiac, Thoracic, Transplantation and Vascular Surgery, Leibniz Research Laboratories for Biotechnology and Artificial Organs, REBIRTH-Center for Regenerative Medicine, Hannover Medical School, Hannover, Germany

[4] Bosch Institute, Department of Anatomy, University of Sydney, Sydney, Australia

MATERIALS and METHODS

Description of the cell line

Cardiac differentiation and subsequent purification of cardioymyocytes derived from hES3 aMyHCneoPGKhygro cells was performed using established protocols (Xu et al., 2008a; Xu et al., 2008b; Kempf et al., 2014; Schwanke et al., 2014).

PKA treatment

To determine the potential functional effect of phosphorylation of some sarcomeric proteins by protein kinase A (PKA), part of d-hESC-CMs and hvMFs were incubated for 45 min at 20°C with *relaxing-solution* (+PIC, +DTT) containing 285 U/mL PKA (P2645, Sigma-Aldrich, Deisenhofen, Germany). After incubation, samples were rinsed with *relaxing-solution* (+PIC, +DTT) without PKA. The other part of d-hESC-CMs and hvMFs received the same treatment without PKA.

Immunostaining

Double immunostaining with antibodies against αMyHC (anti-α-myosin, rabbit, polyclonal, BioGenes, Berlin, Germany) and βMyHC (anti-β-myosin, mouse, monoclonal, Sigma-Aldrich, Deisenhofen, Germany) isoforms of d-hESC-CMs or 5 µm-cryosections from human ventricles was performed as in (Weber et al., 2016). Double immunostaining with antibodies against MLC-2v (rabbit anti-MLC-2v, polyclonal, 10906-1-AP, Acris, Proteintech, Göttingen, Germany) and MLC-2a (mouse anti-MLC-2a, monoclonal, SYSY 311011, Synaptic Systems, Göttingen, Germany) isoforms was performed as in (Weber et al., 2016). Secondary antibodies were the same as in immunostaining against αMyHC and βMyHC (Weber et al., 2016): goat anti-rabbit Alexa Fluor 488 (polyclonal, A11008, Thermo-Fisher, Massachusetts, USA) and goat anti-mouse TRITC (polyclonal, T5393, Sigma-Aldrich, Deisenhofen, Germany), respectively. Single immunostaining was performed with unfixed d-hESC-CMs by incubation for 1h (~20°C) in *relaxing-solution* (+PIC) with the primary antibody, either rabbit anti-αMyHC or mouse anti-α-actinin (monoclonal, Sigma-Aldrich, Deisenhofen, Germany). Then, d-hESC-CMs were rinsed twice with *relaxing-solution* (+PIC) and incubated for 1 h (~20°C) with the rabbit or mouse TRITC-labeled secondary antibody (Sigma-Aldrich, Deisenhofen, Germany), respectively.

Analysis of sarcomeric proteins

Sarcomeric protein analysis was performed either on plated d-hESC-CMs, which were scraped off from the glass coverslips, or on the myofibrillar pellet of adult human ventricular, atrial (Weber et al., 2016) or skeletal (*M. gastrocnemius*) muscle samples. Protein analysis required a larger amount of d-hESC-CMs than for micromechanical experiments. Therefore, the largest batches of hESC-CMs plated for 35, 37 and 42 days were used. All myocyte/muscle samples were demembranated with detergent (0.5% Triton-X-100).

SDS-page gels for detection of sarcomeric proteins ranging from myosin heavy chains [8% SDS gel, with 5% glycerol (Talmadge and Roy, 1993)] to myosin light chains (10% or 12% SDS Gel) and for detection of βMyHC and αMyHC isoforms were previously described (Kraft et al., 2013). Identity of the sarcomeric proteins and their isoforms was either confirmed by western blotting using primary antibodies against: myosin binding protein C (cMyBP-C; Santa Cruz Clone G7, Texas, USA), troponin T (TnT; Abcam Clone TT98, Cambridge, UK), troponin I (TnI; Meridian Clone B2541M, Memphis, Tennessee, USA), tropomyosin (Tm; Sigma Clone TM311, Deisenhofen, Germany), and essential light chain (MLC-1; MA5-15514, Thermo-Fisher, Massachusetts, USA), and/or by the size determined using a protein ruler (PageRuler, Thermo-Fischer, Massachusetts, USA) and Colormarker (BioRad Precision, plus Protein Standard for Westernblot, München, Germany), and/or by direct comparison to the known protein isoform type found in human ventricle, atrium and gastrocnemius muscles. For immunoblot analysis, conjugated secondary antibodies and methodological details were previously explained in (Weber et al., 2016). ProQ Diamond phospho-staining (4-15% gel, Biorad Criterion, München, Germany) and Sypro Ruby-staining (S12000, Thermo-Fisher, Massachusetts, USA) were performed as in (Zaremba et al., 2007). Silver-staining was performed based on a previous description (Heukeshoven and Dernick,

1985). Intensity of some protein isoform bands was assessed with LAS4000 software (GE Healthcare, Solingen, Germany) and analyzed with Totallab TL100 software (Nonlinear Dynamics Ltd. Newcastle, UK).

Micromechanical setup

A single d-hESC-CM or a small hvMFs-bundle was mounted between the tips of a piezo-driven (P-841.20, Physik Instrumente, Waldbronn, Germany) stiff needle (AMS5775, Science Products GmbH, Hofheim, Germany) and a nN-sensitive force probe. d-hESC-CMs and hvMFs were firmly adherent to the tips pre-coated with a 50% (v/v) mixture of flowable silicone rubber (3140 RTV, Dow Corning, Wiesbaden, Germany) with 1% nitrocellulose. As force sensor, we used uncoated cantilevers SD-ZEIL-10 (stiffness 1.2-1.8 nN/nm; NanoAndMore GmbH, Wetzlar, Germany). Force calibration was done after each analyzed sample. For force measurements, a single d-hESC-CM (videos 2,3) or hvMFs-bundle (video 4) was exposed to laminar solution flows of either *relaxing-solution* or to *Ca^{2+}-activating-solution*. Solution change was induced rapidly (10 ms) by the movement of a theta-style micropipette (TGC150-15, Clark Electromedical Instruments, Reading, UK) driven by a piezo-actuator (P289.40, Physik Instrumente, Waldbronn, Germany) as previously described (Colomo et al., 1998; Stehle et al., 2002).

Online supplementary material

A single spontaneously contracting-relaxing intact hESC-CM in cell culture medium (37°C) on a laminin-coated cover-slip is shown in video 1 (VIDEO 1.AVI). Contracting-relaxing (15°C) single demembranated d-hESC-CMs are shown in video 2 (immunostained against α-actinin at Z-disks) and in video 3 (not immunostained), and a contracting-relaxing small hvMFs-bundle (not immunostained) in video 4: VIDEO 2.AVI (corresponding to **Figures 1B$_{1\text{-}3}$**), VIDEO 3.AVI (corresponding to **Figure 1A$_1$**), and VIDEO 4.AVI (corresponding to **Figure 1A$_2$**), respectively. Video 5 (VIDEO 5.AVI) is shown as a proof of principle supporting the estimation of the thickness of myofibrils within d-hESC-CMs (see “Materials and Methods”). Video 1 was taken in phase contrast (PhC), video 2 in fluorescence (FL), videos 3 and 4 in bright field (BF), and video 5 in an alternate manner: PhC, FL and PhC + FL.

RESULTS - Figures and Legends

Figure S1

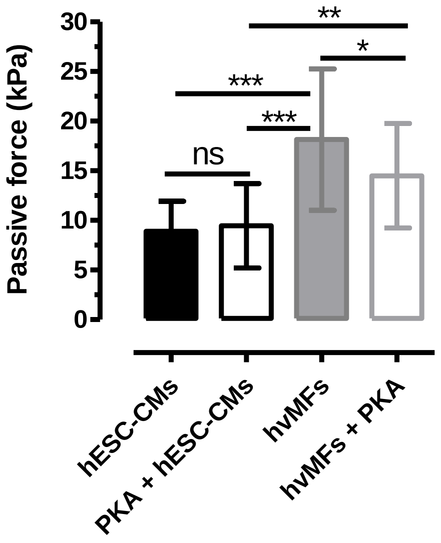

Passive force (F_{pass}) determined before Ca^{2+}-activation (as shown in **Figure 1C**) for myofibrillar bundles within d-hESC-CMs (black bars) and for hvMFs-bundles (grey bars), which were treated (open bars) or not treated (filled bars) with PKA. Note, myofibrils of d-hESC-CMs were more compliant (smaller F_{pass}) than ventricular myofibrils and irresponsive to PKA-treatment. Values for d-hESC-CMs: F_{pass} = 8.9 ± 3.0 kPa (-PKA; n = 12) and F_{pass} = 9.5 ± 4.3 kPa (+PKA; n = 14); for hvMFs: F_{pass} = 18.1 ± 7.1kPa (-PKA; n = 25) and F_{pass} = 14.5 ± 5.3kPa (+PKA; n = 33); *, p = 0.03; **, p = 0.003; ***, p = 0.0002.

Figure S2

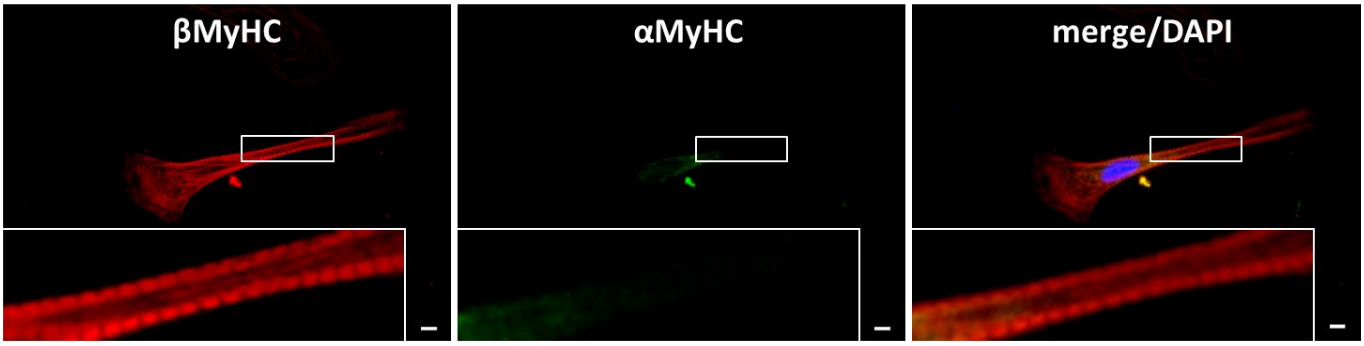

Single d-hESC-CMs double immunostained against βMyHC (red fluorescence) and αMyHC (green fluorescence); blue, DAPI for staining of nuclei. Scale bars = 10 µm. Insets represent the digital zoom of the selected regions. In the αMyHC staining, green fluorescence is very weak compared to red fluorescence and is essentially not sarcomere specific.

Figure S3

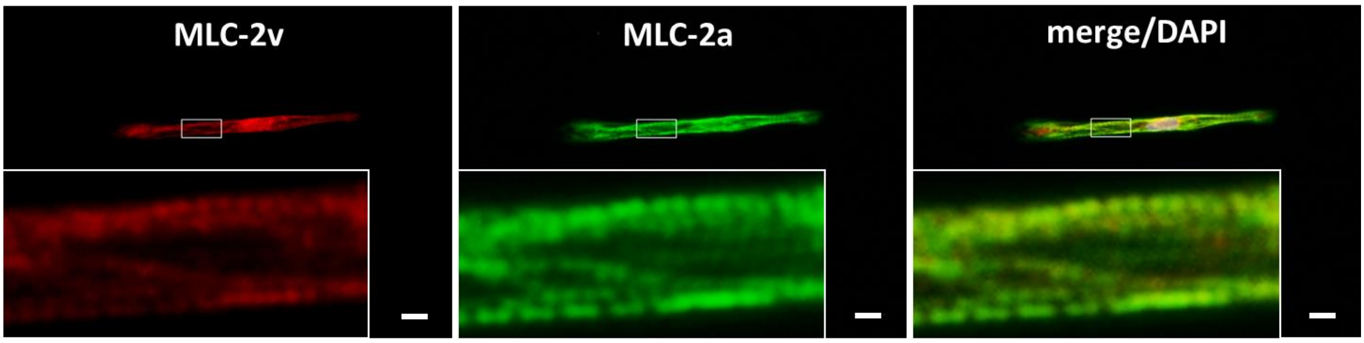

Single d-hESC-CMs double immunostained against MLC-2v (red fluorescence) and MLC-2a (green fluorescence) showing expression of both MLC-2 isoforms in the sarcomeres; blue, DAPI for staining of nuclei. Scale bars = 10 µm. Insets represent the digital zoom of the selected regions.

Figure S4

Western blots

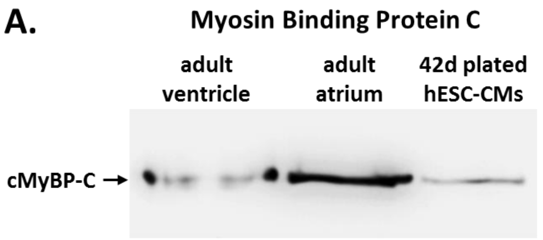

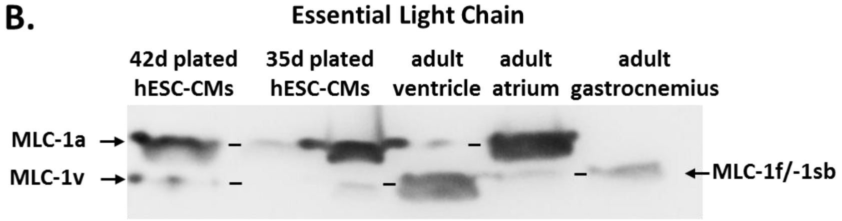

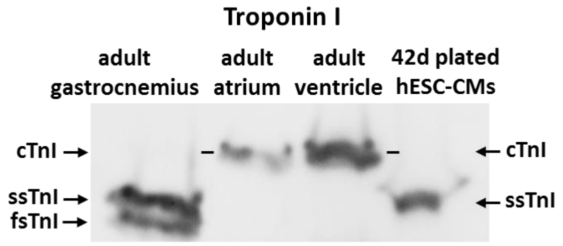

Sypro Ruby - staining

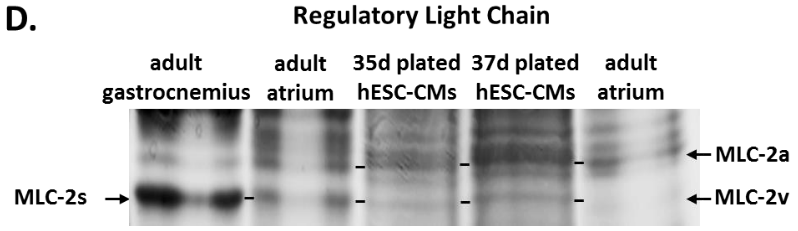

(A-C) Western Blot analysis showing the presence of the following sarcomeric protein isoforms in d-hESC-CMs plated for either 42 or 35 days: **(A)** cMyBP-C (as detected in adult human ventricular and atrial samples); **(B)** MLC-1a and MLC-1v isoforms of the essential light chains (MLC-1a predominates); **(C)** Slow skeletal TnI isoform (ssTnI); Note, cardiac TnI isoform (cTnI) was detected only in the adult ventricular and atrial samples; **(D)** Sypro ruby staining gel showing the presence of both MLC-2a and MLC-2v isoforms of the regulatory light chain in hESC-CMs, while MLC-2a was the predominant isoform; Note, one atrial sample (lane 2) contained a higher percentage of MLC-2v isoform used to identify its position in hESC-CMs.

REFERENCES

Colomo, F., Nencini, S., Piroddi, N., Poggesi, C., and Tesi, C. (1998). Calcium dependence of the apparent rate of force generation in single striated muscle myofibrils activated by rapid solution changes. Adv Exp Med Biol 453, 373-381; discussion 381-372. doi: 10.1007/978-1-4684-6039-1_42.

Heukeshoven, J., and Dernick, R. (1985). Characterization of a solvent system for separation of water-insoluble poliovirus proteins by reversed-phase high-performance liquid chromatography. J Chromatogr 326, 91-101. doi: 10.1016/S0021-9673(01)87434-3.

Kempf, H., Olmer, R., Kropp, C., Ruckert, M., Jara-Avaca, M., Robles-Diaz, D., et al. (2014). Controlling expansion and cardiomyogenic differentiation of human pluripotent stem cells in scalable suspension culture. Stem Cell Reports 3(6), 1132-1146. doi: 10.1016/j.stemcr.2014.09.017.

Kraft, T., Witjas-Paalberends, E.R., Boontje, N.M., Tripathi, S., Brandis, A., Montag, J., et al. (2013). Familial hypertrophic cardiomyopathy: functional effects of myosin mutation R723G in cardiomyocytes. J Mol Cell Cardiol 57, 13-22. doi: 10.1016/j.yjmcc.2013.01.001.

Schwanke, K., Merkert, S., Kempf, H., Hartung, S., Jara-Avaca, M., Templin, C., et al. (2014). Fast and efficient multitransgenic modification of human pluripotent stem cells. Hum Gene Ther Methods 25(2), 136-153. doi: 10.1089/hgtb.2012.248.

Stehle, R., Kruger, M., Scherer, P., Brixius, K., Schwinger, R.H., and Pfitzer, G. (2002). Isometric force kinetics upon rapid activation and relaxation of mouse, guinea pig and human heart muscle studied on the subcellular myofibrillar level. Basic Res Cardiol 97 Suppl 1, I127-135. doi: 10.1007/s003950200041.

Talmadge, R.J., and Roy, R.R. (1993). Electrophoretic separation of rat skeletal muscle myosin heavy-chain isoforms. J Appl Physiol (1985) 75(5), 2337-2340. doi: 10.1152/jappl.1993.75.5.2337.

Weber, N., Schwanke, K., Greten, S., Wendland, M., Iorga, B., Fischer, M., et al. (2016). Stiff matrix induces switch to pure beta-cardiac myosin heavy chain expression in human ESC-derived cardiomyocytes. Basic Res Cardiol 111(6), 68. doi: 10.1007/s00395-016-0587-9.

Xu, X.Q., Graichen, R., Soo, S.Y., Balakrishnan, T., Rahmat, S.N., Sieh, S., et al. (2008a). Chemically defined medium supporting cardiomyocyte differentiation of human embryonic stem cells. Differentiation 76(9), 958-970. doi: 10.1111/j.1432-0436.2008.00284.x.

Xu, X.Q., Zweigerdt, R., Soo, S.Y., Ngoh, Z.X., Tham, S.C., Wang, S.T., et al. (2008b). Highly enriched cardiomyocytes from human embryonic stem cells. Cytotherapy 10(4), 376-389. doi: 10.1080/14653240802105307.

Zaremba, R., Merkus, D., Hamdani, N., Lamers, J.M., Paulus, W.J., Dos Remedios, C., et al. (2007). Quantitative analysis of myofilament protein phosphorylation in small cardiac biopsies. Proteomics Clin Appl 1(10), 1285-1290. doi: 10.1002/prca.200600891.
